# Protein-ligand binding affinity determination by the waterLOGSY method: An optimised approach considering ligand rebinding

**DOI:** 10.1038/srep43727

**Published:** 2017-03-03

**Authors:** Renjie Huang, Arnaud Bonnichon, Timothy D. W. Claridge, Ivanhoe K. H. Leung

**Affiliations:** 1 School of Chemical Sciences, The University of Auckland, Private Bag 92019, Victoria Street West, Auckland 1142, New Zealand; 2 Department of Chemistry, University of Oxford, Chemistry Research Laboratory, 12 Mansfield Road, Oxford OX1 3TA, United Kingdom; 3 Université D’Auvergne, 49 Boulevard François-Mitterrand, CS 60032, Clermont Ferrand 63001, Cedex 1, France

## Abstract

WaterLOGSY is a popular ligand-observed NMR technique to screen for protein-ligand interactions, yet when applied to measure dissociation constants (*K*_D_) through ligand titration, the results were found to be strongly dependent on sample conditions. Herein, we show that accurate *K*_D_s can be obtained by waterLOGSY with optimised experimental setup.

Modern drug discovery is often based on finding novel small molecule inhibitors that can bind disease-related proteins and inhibit their biological activities and interactions^1^,^2^,^3^. Understanding how small molecule inhibitors bind their target proteins is therefore one of the major focuses of biochemical and medicinal research, and knowing the strength of this interaction and is an important step in any drug discovery programme as it enables ranking of inhibitors based on their binding affinities. A common way to quantify the strength of protein-ligand interactions is by the dissociation constant (or binding constant, *K*_D_).

Nuclear magnetic resonance (NMR) spectroscopy is an established method for the screening of protein ligands and for the quantification of protein-ligand binding^4^,^5^,^6^,^7^,^8^,^9^,^10^. Broadly, there are two NMR-based protocols to study these binding interactions. The first involves the observation of proteins that are isotopically labelled, either in the backbone amides (e.g. ^15^N) or with unnatural amino acids (e.g. fluorinated analogues)^7^,^11^,^12^. With these, binding constants can be measured in a ligand titration experiment by following peak intensity changes or chemical shift perturbations of the labelled amino acid resonances^4^,^5^,^6^. A second class of NMR techniques utilises the observation of ligand resonances^8^,^9^,^10^. The ligands of interest are usually small molecules with molecular weight <1 kDa enabling the methods to exploit the large differences in size between the protein and ligand. These ligand-observe experiments may be further subdivided into two categories. The first involves observing changes in NMR parameters of the ligand resonances directly, such as relaxation times, linewidths and chemical shifts. The second category involves the observation of ligand binding indirectly through the nuclear Overhauser effect (NOE). The water-ligand observed via gradient spectroscopy (waterLOGSY) experiment belongs to this category.

WaterLOGSY is a method initially designed for the screening of protein ligands from a mixture of potential binders^13^,^14^. In the waterLOGSY experiment, compounds that bind to the target protein often give positive resonances (i.e. have the same sign as protein resonances), and compounds that do not interact with the protein usually give negative resonances (Supplementary Fig. S1). The experiment relies on fast dissociation of bound ligands to carry into the free state magnetisation originating from irradiated solvent water that has been transmitted via the protein complex, and so typically works best for weak binding systems, such as those with dissociation constants in the high μM to mM region^15^.

WaterLOGSY may, in principle, be applied to measure *K*_D_s through ligand titration experiments^14^. The observed waterLOGSY signal of a binding ligand is an average between the NOE that arises for the free population and that which arises for the bound population, which have opposing signs. Corrections to the observed waterLOGSY signals are needed to be made so that the binding isotherm only reflects the bound ligand population. This can be achieved by conducting a control titration that contains only the ligand (i.e. in the absence of the protein; Supplementary Fig. S2). Dalvit *et al*. demonstrated the use of waterLOGSY to obtain *K*_D_ with human serum albumin (HSA) and L-tryptophan. By titrating L-tryptophan (50 μM to 600 μM) into a fixed concentration (10 μM) of HSA, a *K*_D_ of 290 μM was obtained^14^. The measured *K*_D_ was in agreement with reported binding constants that were obtained by NMR and also other biophysical methods^16^,^17^. The use of waterLOGSY to measure *K*_D_s has since been applied by several research groups^18^,^19^,^20^.

There is, however, a caveat about the accuracy of such titration experiments as it was discovered that the observed *K*_D_ values (*K*_D_^obs^) yielded by waterLOGSY may be influenced by experimental conditions^21^. Fielding *et al*. demonstrated that *K*_D_^obs^ values became greater with increasing protein concentrations by conducting a series of L-tryptophan titration experiments to varying concentrations of bovine serum albumin (BSA) ranging from 15 to 75 μM. In this, a *K*_D_^obs^ of 230 μM was obtained with 15 μM BSA, but a ten-fold greater value of 3.0 mM was obtained with 75 μM BSA. The affinity between a protein and a ligand ought to be independent of the protein concentration, thus the *K*_D_^obs^ values obtained by waterLOGSY at higher protein concentrations were overestimated.

A similar influence of experimental conditions on the *K*_D_^obs^ values has been reported for saturation transfer difference (STD) NMR^22^, another NOE-based ligand observe NMR technique. Conceptually STD NMR is similar to waterLOGSY, with magnetisation transferred via NOE from the saturated protein to the bound ligands^23^. It was observed that *K*_D_^obs^s obtained from STD NMR experiments vary significantly with saturation time (i.e. time allowed for the magnetisation to transfer), and accurate *K*_D_ values could only be determined when the experiments were conducted at a saturation times that were close to zero^22^. The deviation of *K*_D_^obs^ to the ‘true’ *K*_D_ value was attributed to the rebinding of partially saturated ligands to the protein during the protein irradiation period.

Herein, we report on systematic investigations of the use of waterLOGSY to determine protein-ligand binding constants. Our data show that *K*_D_^obs^ values obtained by waterLOGSY titration experiments were indeed strongly dependent on solution conditions, leading to an overestimation of *K*_D_ values. We further show that accurate binding constants can be determined if the likelihood of ligand rebinding is minimised through use of appropriate experimental conditions.

## Results and Discussion

We hypothesised that the deviation from the ‘true’ *K*_D_ of *K*_D_^obs^ obtained from a waterLOGSY titration experiment originates from a similar mechanism to that reported for STD NMR, namely ligand rebinding during the mixing period^22^. In this time there exists the probability that a ligand that is already carrying saturation from a binding event may re-enter the binding site before its perturbed magnetisation has fully relaxed back to equilibrium^22^. Consequently, the observed waterLOGSY signals become reduced in intensity and the resultant binding titration profile does not accurately reflect the 1:1 binding model that was originally assumed^14^, yielding overestimated dissociation constants. The probability of such fast rebinding processes is increased at higher protein concentrations^22^, suggesting the greatest perturbation from the ‘true’ *K*_D_ value may be anticipated for systems with high protein concentrations.

In order to validate this proposal, we repeated the observations made by Fielding *et al*., choosing the same model system of serum albumin and L-tryptophan as used by Dalvit *et al*. and Fielding *et al*.^14^,^21^. The reported *K*_D_ values for L-tryptophan and HSA range from ~100 μM to ~250 μM^16^,^17^,^21^. As serum albumins are known to have multiple low affinity L-tryptophan binding sites^24^,^25^, the standard model that assumes binding to a single site was not used because this does not take into account non-specific binding^4^,^5^,^6^,^26^. Taira and Terada proposed an alternative model by assuming one high affinity binding site and an unlimited number of low affinity non-specific sites, which they tested with several serum albumin-ligand systems^27^. We found that this model gave better non-linear curve fitting to our titration data with both HSA and BSA than the standard 1:1 binding model (Supplementary Fig. S3), so was applied throughout this study. The H-δ signal of the bicyclic ring of L-tryptophan was integrated for this study because it is a singlet and gives the highest intensity (Supplementary Fig. S4).

**Figure 1:**
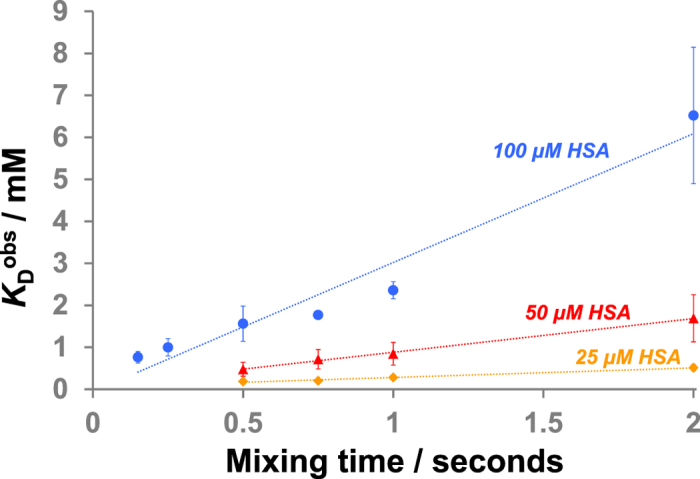
The correlation between observed *K*_D_ (*K*_D_^obs^), mixing time and HSA concentration for L-tryptophan binding (reported *K*_D_ ~200 μM; see text). Error bars show standard errors from three separate experiments. Dotted lines are added to aid visualisation.

We first measured the *K*_D_s of L-tryptophan to HSA at a long mixing time (the period in which magnetisation transfer onto the ligand occurs) of 2.0 seconds. Three different HSA concentrations were used and in agreement with previous reports^21^, we observed that the *K*_D_^obs^ increases as the protein concentration increases, ranging from 510 μM to 6.5 mM (Table 1, Fig. 1 and Supplementary Figs S5–S7). Similar phenomena were observed when STD NMR was applied for direct *K*_D_ measurements, in which the variation in *K*_D_^obs^ was attributed to the rebinding of partially-saturated ligands to the protein during the saturation time period^22^.

We conducted similar experiments with BSA, observing that at 20 μM, the *K*_D_^obs^ for L-tryptophan was ~120 μM, but this increased to ~1.5 mM when the titration was conducted with 75 μM BSA (Supplementary Figs S8 and S9). These results confirmed previous observations that the use of waterLOGSY to measure protein-ligand *K*_D_s may lead to their overestimation^21^. Deviation from the true *K*_D_ value was again most severe at high protein concentrations, which is consistent with our proposal that this inaccuracy originates from rebinding to the protein of partially saturated ligands^22^.

We also sought to investigate the influence on measured *K*_D_s of waterLOGSY mixing times. It was envisaged that at short mixing times there will be less opportunity for partially saturated ligands to rebind, and therefore the *K*_D_^obs^ would approach the true *K*_D_ value. Six different mixing times were used ranging from 0.15 seconds to 2.0 seconds with a HSA concentration fixed at 100 μM. Our results showed that *K*_D_^obs^ deviated strongly from the true *K*_D_ value even with modest mixing times (e.g. <1 second). At very short mixing time (0.15 seconds), a *K*_D_^obs^ of 760 μM was obtained, but a linear increase up to 6.5 mM was observed as the mixing times became longer (Table 1, Fig. 1 and Supplementary Figs S7, S10–S14). These results show that under conditions that are typically employed in waterLOGSY experiments (mixing time of ~1 second), an overestimation of *K*_D_ may result, so limiting the applicability of waterLOGSY for quantitative ligand binding analyses.

High protein concentrations may encourage the unwanted rebinding of previously saturated ligands, and in accordance with this, we found that the deviation of *K*_D_^obs^ with mixing time was less apparent when the protein concentration was reduced. When L-tryptophan titrations were conducted with 50 μM HSA and a short 0.5 s mixing time, a *K*_D_^obs^ of 480 μM was obtained (approximately twice the reported *K*_D_ value), in contrast to 1.5 mM observed with 100 μM protein at this mixing time. The increase in *K*_D_^obs^ as a function of mixing time was also less significant at reduced HSA concentrations (Table 1 and Fig. 1); it was not possible to measure meaningful *K*_D_^obs^ at mixing time less than 0.5 seconds due to poor signal-to-noise (Supplementary Figs S6, S15–S17).

Most significantly, there was relatively little variation in the *K*_D_^obs^ values with mixing times between 0.5 and 1.0 second when the L-tryptophan titrations were conducted with 25 μM HSA, ranging from 190 μM up to 280 μM (Table 1, Fig. 1 and Supplementary Figs S5, S18–S20). These results suggest that the derivations of *K*_D_^obs^ from the ‘true’ *K*_D_ value as a function of mixing times will reduce when a lower protein concentration is used. They also suggest that it may be possible to obtain an accurate *K*_D_ value even with a long mixing time (e.g. 1.0 second) if the experiment was conducted with a sufficiently low protein concentration.

**Figure 2:**
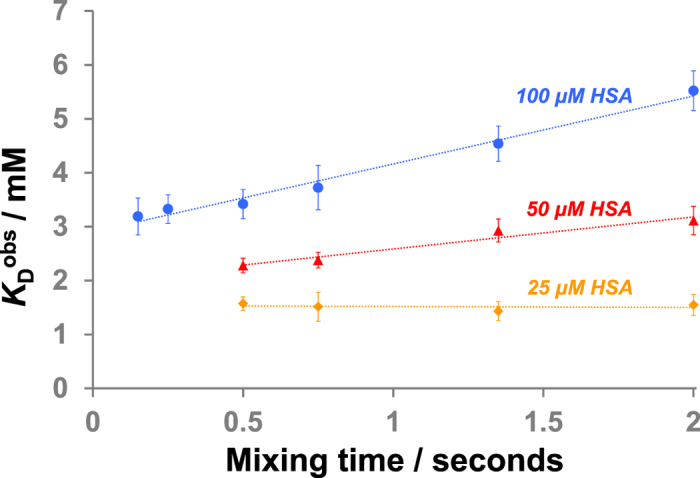
The correlation between observed *K*_D_ (*K*_D_^obs^), mixing time and HSA concentration for caffeine binding (reported *K*_D_ ~1.5 mM). Error bars show standard errors from three separate experiments. Dotted lines are added to aid visualisation.

To explore this notion further, we selected caffeine as an alternative, weaker binding ligand with a reported *K*_D_ value for binding to HSA varying between 0.9 mM and 1.5 mM^21^,^28^,^29^,^30^. Similar to L-tryptophan, there also exists multiple weak non-specific caffeine binding sites on HSA^28^,^29^,^30^. Titration experiments were again conducted with varying mixing times (0.15 seconds to 2.0 seconds) and varying protein concentrations (25 μM, 50 μM and 100 μM). When the caffeine titration was conducted with 100 μM HSA, even at a very short mixing time (0.15 seconds), an erroneous *K*_D_^obs^ of 3.2 mM was determined, and we again observed a progressive increase in the value with longer mixing times (Table 2, Fig. 2 and Supplementary Figs S21–S26). Similar to L-tryptophan, these deviations of *K*_D_^obs^ from the true *K*_D_ value with different mixing times decreased when the titrations were conducted at lower protein concentrations, as observed in similar work using STD NMR for direct *K*_D_ measurements^22^. In fact, when the experiments were performed with 25 μM HSA, no variation of *K*_D_^obs^ was observed as a function of mixing time and all experiments yielded a *K*_D_^obs^ of ~1.5 mM, consistent with reported data (Fig. 2 and Supplementary Figs S27–S34). This again indicates that it is possible to obtain an accurate dissociation constant by waterLOGSY titration provided a sufficiently low protein concentration is used, and suggests a means of determining whether the protein concentration used was appropriately ‘low’. Conducting titration experiments using (at least) two mixing times (e.g. 0.5 and 1.0 second) should yield similar *K*_D_ values in the optimised situation, which reflect the true dissociation constant. It is also worth noting that with L-tryptophan at 25 μM HSA concentration, meaningful *K*_D_^obs^ values could be obtained with a mixing time of up to 1.0 second, whilst for caffeine at 25 μM HSA concentration, it was possible to obtain meaningful *K*_D_^obs^ values even at a very long mixing time (e.g. 2.0 seconds). The relationship between *K*_D_^obs^ and mixing time is likely complex due to multiple competing factors including (but not limited to) the extent of ligand rebinding and ligand dissociation rates. It is therefore advisable not to conduct quantitative measurements at excessively long mixing times ( >1.0 sec) to avoid any derivation from the true *K*_D_ value.

Finally, in order to demonstrate that this protocol is also applicable to 1:1 protein-ligand system in the absence of multiple non-specific binding sites, the binding affinity of a boronic acid with α-chymotrypsin was measured. α-Chymotrypsin is a serine protease known to bind boronic acids as a reversible covalent complex through the formation of a tetrahedral adduct via its nucleophilic serine residue (Supplementary Fig. S35)^31^. 3-Fluorophenylboronic acid (Supplementary Fig. S35) was chosen as the model ligand system because its binding constant to α-chymotrypsin could be determined independently using ^19^F NMR spectroscopy.

Thus, by titrating 3-fluorophenylboronic acid to 100 μM α-chymotrypsin and monitoring changes in the boronic acid’s ^19^F chemical shift, a *K*_D_ of 630 μM was determined (Supplementary Fig. S36). WaterLOGSY was then conducted with varying mixing times (0.5 to 2.0 seconds) and varying protein concentrations (10 μM, 25 μM, 50 μM and 100 μM; Supplementary Fig. S37). Similar to our observations with the serum albumins, deviations in *K*_D_^obs^ were observed at high α-chymotrypsin concentrations. For example, millimolar *K*_D_^obs^ values were obtained when the titrations were conducted with 50 μM and 100 μM α-chymotrypsin concentrations (Supplementary Figs S38–S49). However, when the titrations were conducted at low α-chymotrypsin concentration (10 μM), the *K*_D_^obs^ values obtained at both short and long mixing times were similar (~630, ~780 and ~700 μM at 0.5, 1.0 and 2.0 s mixing time respectively), and in agreement with the *K*_D_ value obtained by ^19^F NMR (630 μM), again suggesting that an accurate *K*_D_ value may be obtained by waterLOGSY when a ‘low’ protein concentration is used.

Taken together, these results confirmed previous observations that measured *K*_D_ values are influenced directly by the protein concentrations employed in ligand titrations^21^. We hypothesise that this is likely due to the rebinding of ligands that carry the negative NOE obtained from a previous binding event, as it was proposed for STD NMR measurements, another NOE-based method for the measurement of protein-ligand interactions^22^. These studies show that the deviation of *K*_D_^obs^ from the true *K*_D_ value is particularly severe at high protein concentrations and long mixing times, in agreement with this proposal. They also showed that it is possible to eliminate this detrimental influence by using a ‘low’ protein concentration, which may be determined by conducting titration experiments with (at least) two different mixing times. Any increase in measured *K*_D_ with mixing time would suggest the *K*_D_ values determined may be higher than the true value, at least placing an upper limit on the dissociation constant. There already exist many biophysical techniques, including NMR, able to measure binding affinities, although, most are designed to measure relatively strong protein-ligand binding interactions. If *K*_D_ determination is necessary for a weak protein-ligand binding system (e.g. during a fragment screen), the choice of biophysical techniques for such a measurement is limited. Whilst the use of waterLOGSY to measure *K*_D_ may not be required on a regular basis, we believe it fills an important gap in the arsenal of techniques available to cover the whole range of ligand binding affinities and the protocols suggested here should aid in its application, or at least help avoid its inappropriate use.

## Methods

### Materials

Unless otherwise stated, all chemicals were from Sigma-Aldrich. Tris-d11 was from Cortecnet. D_2_O and DMSO-d6 were from Cambridge Isotope Laboratories. HSA and BSA (both fatty acid free and globulin free, ≥99%) and α-chymotrypsin (from bovine pancreas, Type II, ≥40 units/mg protein) were from Sigma-Aldrich.

### NMR experiments

HSA waterLOGSY experiments were conducted at a ^1^H frequency of 600 MHz using a Bruker Avance spectrometer equipped with a BBI probe. All experiments were conducted at 298 K. 5 mm diameter NMR tubes with a sample volume of 500 μL were used in all experiments. Solutions were buffered using 50 mM sodium phosphate (pH 7.5) dissolved in 90% H_2_O and 10% D_2_O.

BSA waterLOGSY experiments were conducted at a ^1^H frequency of 700 MHz using a Bruker Avance III spectrometer equipped with a TCI cryoprobe. All experiments were conducted at 293 K. 5 mm diameter NMR tubes with a sample volume of 500 μL were used in all experiments. Solutions were buffered using 50 mM Tris-d11 (pH 7.5) and 0.02% NaN_3_ dissolved in 90% H_2_O and 10% D_2_O.

α-Chymotrypsin waterLOGSY experiments were conducted at a ^1^H frequency of 700 MHz using a Bruker Avance III spectrometer equipped with a TCI cryoprobe. All experiments were conducted at 298 K. 5 mm diameter NMR tubes with a sample volume of 500 μL were used in all experiments. Solutions were buffered using 100 mM MES buffer (pH 6.5) dissolved in 90% H_2_O and 10% D_2_O.

α-Chymotrypsin ^19^F experiments were conducted at a ^19^F frequency of 470 MHz using a multinuclear BBFO probe. All experiments were conducted at 298 K. 5 mm diameter NMR tubes with a sample volume of 500 μL were used in all experiments. Solutions were buffered using 100 mM MES buffer (pH 6.5) dissolved in 90% H_2_O and 10% D_2_O. Trifluoroacetone was added to the sample for chemical shift reference at −87 ppm vs CFCl_3_.

WaterLOGSY experiments were conducted using the pulse sequence as described by Dalvit *et al*.^13^,^14^. The pulse tip-angle calibration using the single-pulse nutation method (Bruker *pulsecal* routine) was undertaken for each sample^33^. Typical experimental parameters were as follows: relaxation delay 15 seconds (BBI probe) or 5 seconds (cryoprobe), number of transients 64–256 (BBI probe) or 16 (cryoprobe). Solvent excitation was achieved using a 7.5 ms 180 degree selective *Gaus1_180r.1000 pulse* and water suppression was achieved by the excitation sculpting method using a 2 ms 180 degree selective *Sinc1.1000* pulse at the H_2_O frequency, supplemented with a 4 ms 90 degree selective *Sinc1.1000* water flip-back pulse.

### Dissociation constant determination

*K*_D_^obs^ for HSA were obtained by fitting the binding isotherm with equation (1)^27^:


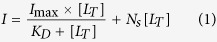


*K*_D_^obs^ for α-chymotrypsin were obtained by fitting the binding isotherm with equation (2)^4^,^5^,^6^:


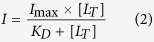


*I* indicates changes in corrected waterLOGSY intensity from the titrations. *I*_*max*_ indicates the maximum intensity change. [*L*_*T*_] is the titrated ligand concentration and *N*_*s*_ is the non-specific term (where applicable). This uses the approximation [*L_T_*] ~ [*L*] (the *free* ligand concentration) which is valid when the ligand is used in large excess over the protein. Curve fitting process was conducted using SigmaPlot 12.5 (Systat Software, USA).

## Additional Information

**How to cite this article:** Huang, R. *et al*. Protein-ligand binding affinity determination by the waterLOGSY method: An optimised approach considering ligand rebinding. *Sci. Rep.* **7**, 43727; doi: 10.1038/srep43727 (2017).

**Publisher's note:** Springer Nature remains neutral with regard to jurisdictional claims in published maps and institutional affiliations.

## Supplementary Material

Supplementary Information

## Figures and Tables

**Table 1:** Observed *K*_D_ (*K*_D_^obs^) values of L-tryptophan binding to HSA (reported *K*_D_ ~200 μM) measured by waterLOGSY titration at varying mixing times and protein concentrations.

Mixing time/s	*K*_D_^obs^/μM		
	25 μM HSA	50 μM HSA	100 μM HSA
0.15	−	−	765 ± 140
0.25	−	−	1000 ± 200
0.5	185 ± 50	475 ± 165	1500 ± 400
0.75	200 ± 45	715 ± 230	1800 ± 100
1	280 ± 40	845 ± 270	2300 ± 200
2	510 ± 80	1700 ± 500	6500 ± 1600

Errors show are standard errors from three separate experiments.

**Table 2:** Observed *K*_D_ (*K*_D_^obs^) values of caffeine binding to HSA (reported *K*_D_ ~1.5 mM) measured by waterLOGSY titration at different mixing times and protein concentrations.

Mixing time/s	*K*_D_^obs^/mM		
	25 μM HSA	50 μM HSA	100 μM HSA
0.15	−	−	3.2 ± 0.3
0.25	−	−	3.3 ± 0.3
0.5	1.6 ± 0.1	2.3 ± 0.1	3.4 ± 0.3
0.75	1.5 ± 0.3	2.4 ± 0.1	3.7 ± 0.4
1.35	1.4 ± 0.2	2.9 ± 0.2	4.5 ± 0.3
2	1.5 ± 0.2	3.1 ± 0.3	5.5 ± 0.4

Errors shown are standard errors from three separate experiments.
