# Supplementary material for: Protein-ligand binding affinity determination by the waterLOGSY method: An optimised approach considering ligand rebinding
Source: Sci Rep. 2017 Mar 3;7:43727. doi: 10.1038/srep43727 (PMC5335602; doi:10.1038/srep43727)
Supplement: Supplementary Information [file srep43727-s1.pdf]

Supplementary information

Protein-ligand binding affinity determination by the waterLOGSY method: An optimised approach considering ligand rebinding.

Renjie Huang,[1] Arnaud Bonnichon,[2,3] Timothy D. W. Claridge,*[,2] Ivanhoe K. H. Leung.*[,1]

1. School of Chemical Sciences, The University of Auckland, Private Bag 92019, Victoria Street West, Auckland 1142, New Zealand.

2. Department of Chemistry, University of Oxford, Chemistry Research Laboratory, 12 Mansfield Road, Oxford OX1 3TA, United Kingdom.

3. Université D'Auvergne, 49 Boulevard François-Mitterrand, CS 60032, 63001 Clermont Ferrand, Cedex 1, France.

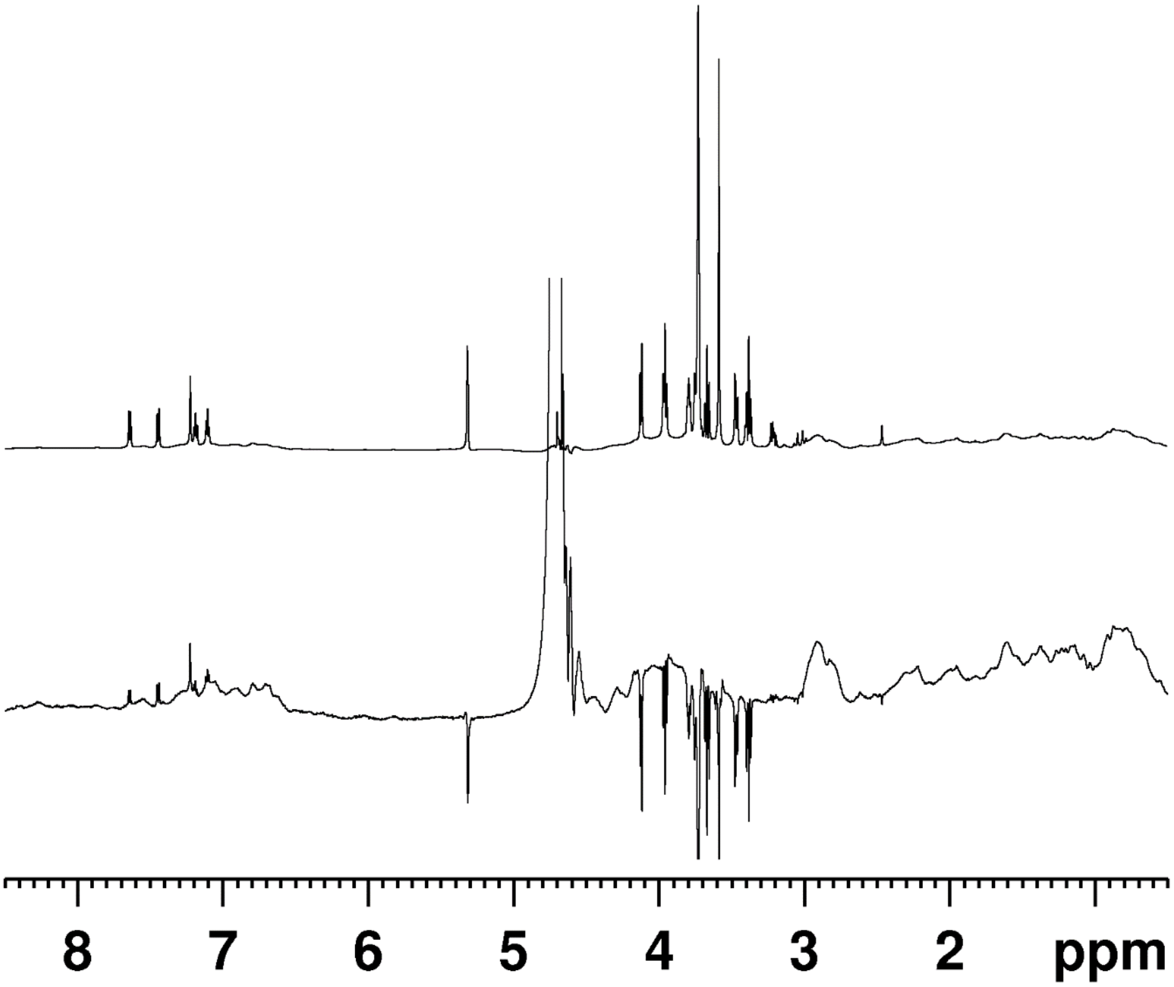

Supplementary Figure S1: A typical waterLOGSY spectrum acquired at 700 MHz (bottom) and its corresponding ^{1}H spectrum (top). The mixture contained 100 μM bovine serum albumin (BSA), 2 mM L-tryptophan (binder) and 2 mM sucrose (non-binder) in 90% H_2O and 10% D_2O. Selective on-resonance irradiation was applied at 4.7 ppm. The mixing time was 1 second and the relaxation delay was 2 seconds. The non-binder exhibits positive NOE, and the protein and the binder exhibit negative NOE. For better visualisation, positive NOE is usually phased negative and negative NOE is phased positive.

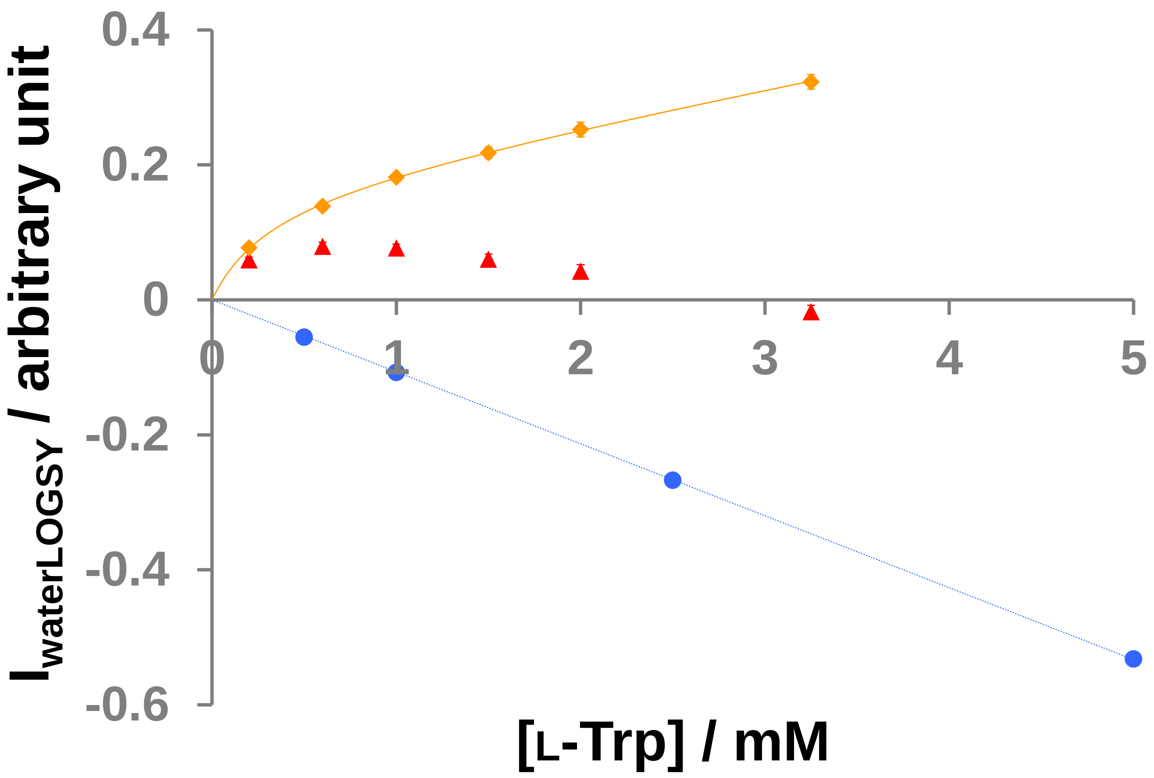

Supplementary Figure S2: The red curve was obtained with waterLOGSY at different ligand concentrations in the presence of the protein (i.e. both positive and negative NOE). The mixture contained 25 μM human serum albumin (HSA) and 0.2–3.25 mM L-tryptophan (binder) in 50 mM sodium phosphate buffer (pH 7.5) at 90% H_2O and 10% D_2O. The blue curve (control) was obtained with waterLOGSY at different ligand concentrations in the absence of the protein (i.e. positive NOE). The orange curve is the corrected waterLOGSY binding isotherm, which is obtained by subtracting the blue curve from the red curve. Selective on-resonance irradiation of water protons was applied at 4.7 ppm. The mixing time was 1 second and the relaxation delay was 15 seconds. The errors shown are the standard error from three separate measurements.

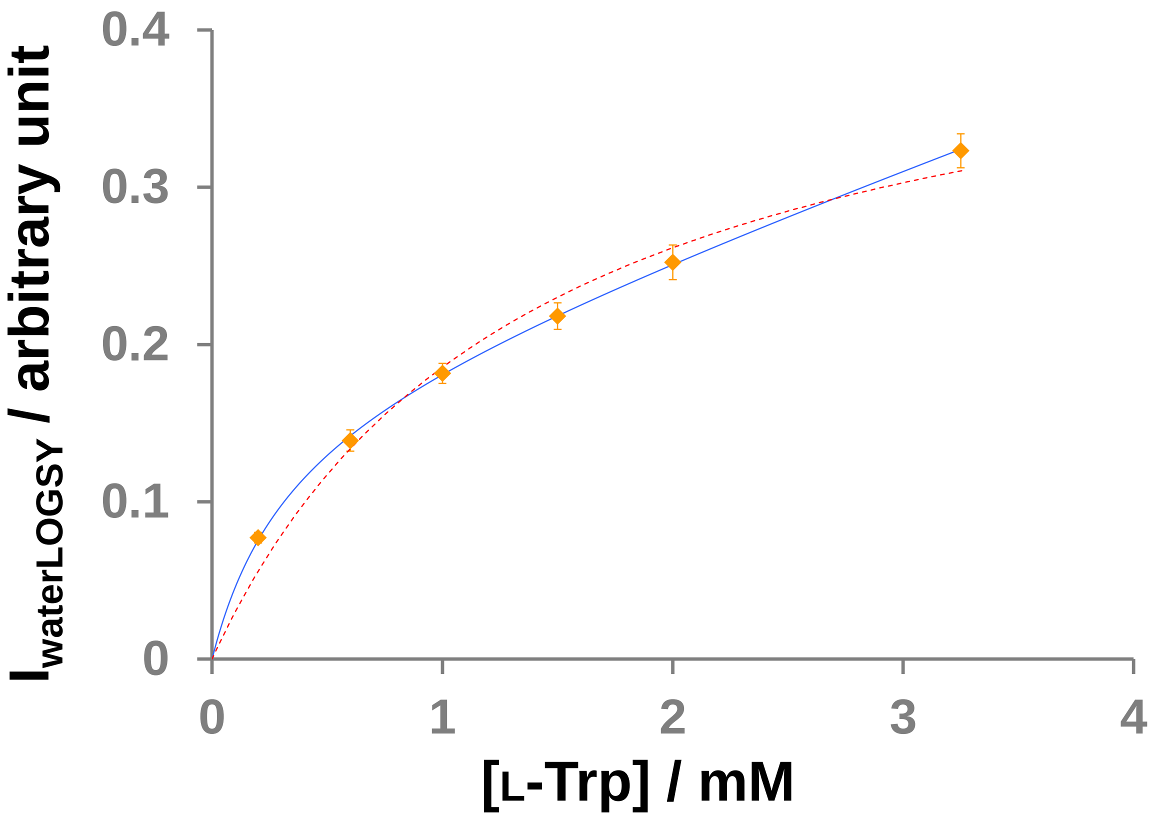

Supplementary Figure S3: Non-linear curve fitting to a corrected waterLOGSY binding isotherm. The system contained 25 μM human serum albumin (HSA) and 0.2–3.25 mM L-tryptophan (binder) in 50 mM sodium phosphate buffer (pH 7.5) at 90% H_2O and 10% D_2O. The blue curve was fitted with the model proposed by Taira and Terada, which takes non-specific binding to serum albumin into account, and the red dotted curve was fitted with the standard 1:1 binding model, which does not take non-specific binding into account. The errors shown are the standard error from three separate measurements.

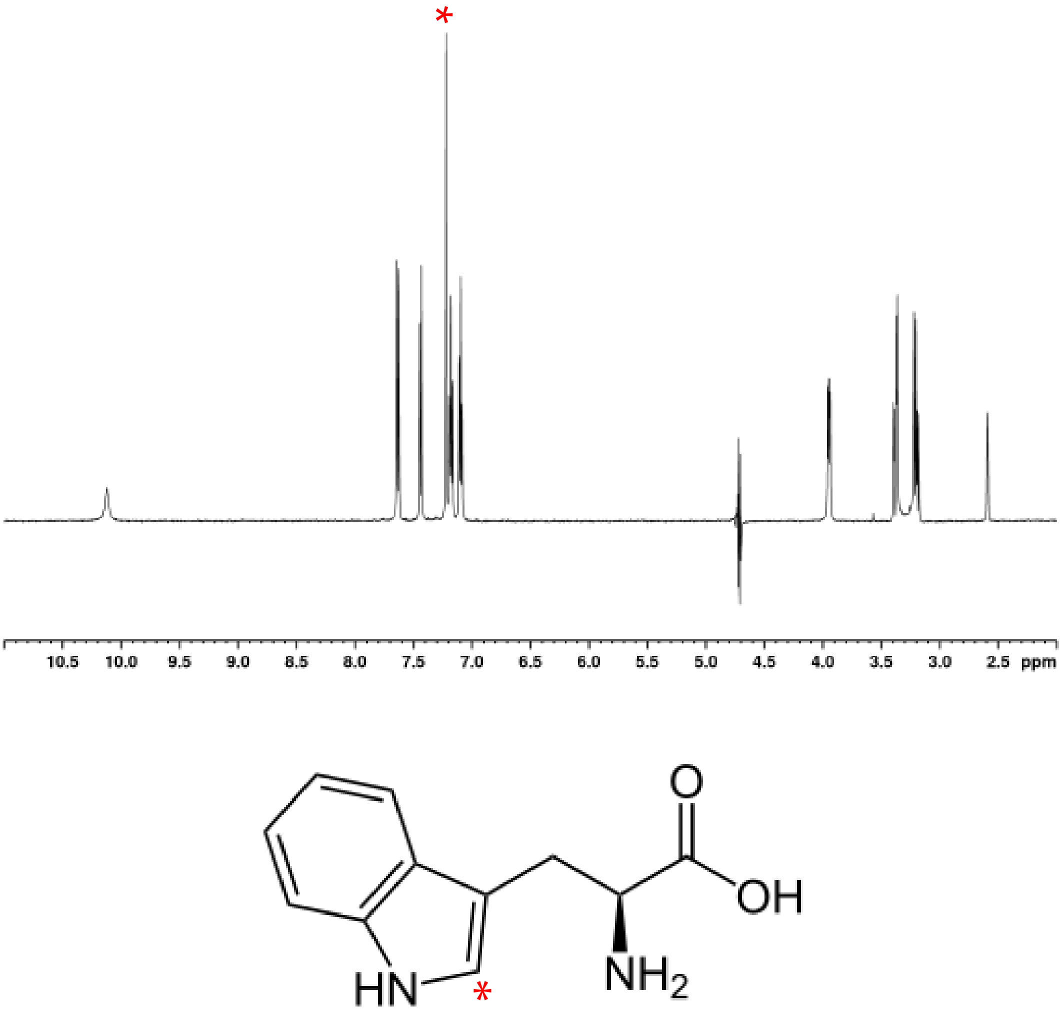

Supplementary Figure S4: H-δ on L-tryptophan (asterisk) is a singlet and has the strongest intensity. It was therefore integrated for all K_D determination in this study.

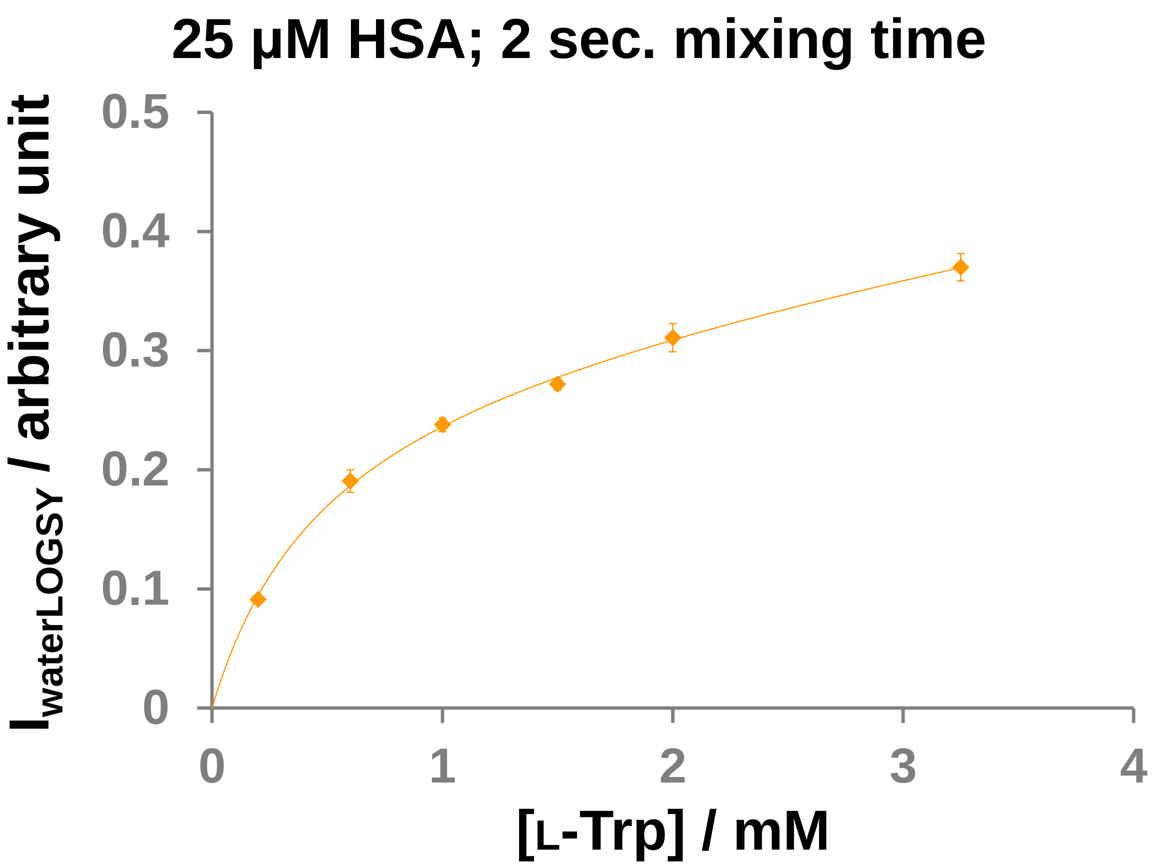

Supplementary Figure S5: Non-linear curve fitting to a corrected waterLOGSY binding isotherm. The mixing time was 2 seconds and the relaxation delay was 15 seconds. The system contained 25 µM human serum albumin (HSA) and 0.2–3.25 mM L-tryptophan (binder) in 50 mM sodium phosphate buffer (pH 7.5) at 90% H_2O and 10% D_2O. The fitted K_D^{obs} was 510 µM ± 80 µM. The errors shown are the standard error from three separate measurements.

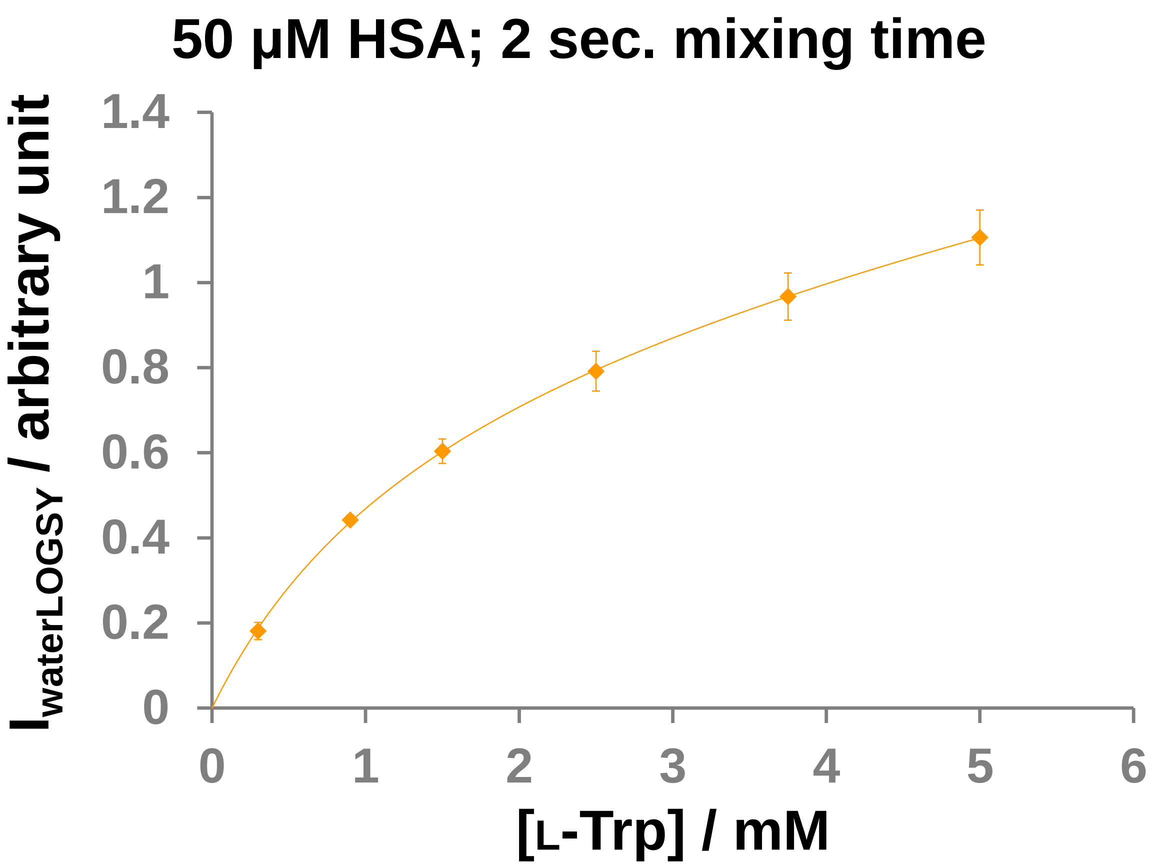

Supplementary Figure S6: Non-linear curve fitting to a corrected waterLOGSY binding isotherm. The mixing time was 2 seconds and the relaxation delay was 15 seconds. The system contained 50 µM human serum albumin (HSA) and 0.3–5.0 mM L-tryptophan (binder) in 50 mM sodium phosphate buffer (pH 7.5) at 90% H_2O and 10% D_2O. The fitted K_D^{obs} was 1.7 mM ± 0.5 mM. The errors shown are the standard error from three separate measurements.

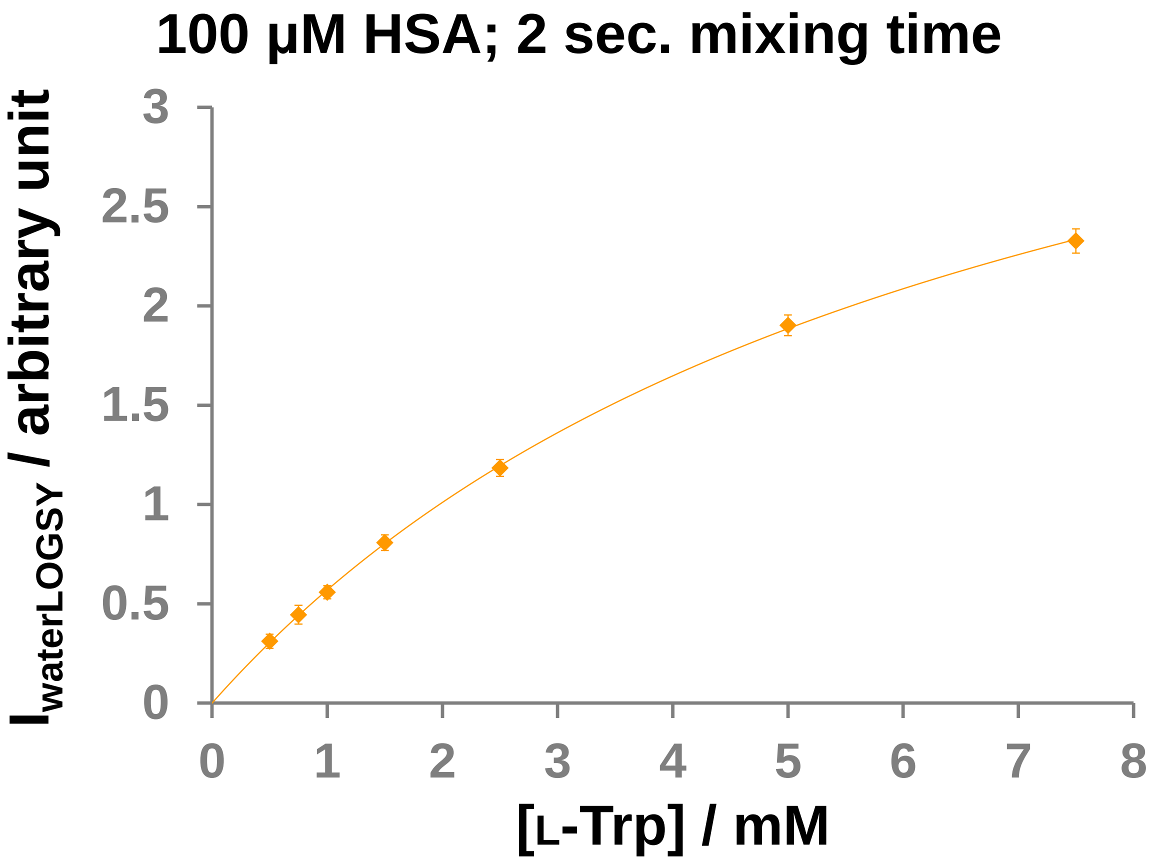

Supplementary Figure S7: Non-linear curve fitting to a corrected waterLOGSY binding isotherm. The mixing time was 2 seconds and the relaxation delay was 15 seconds. The system contained 100 µM human serum albumin (HSA) and 0.5–7.5 mM L-tryptophan (binder) in 50 mM sodium phosphate buffer (pH 7.5) at 90% H_2O and 10% D_2O. The fitted K_D^{obs} was 6.5 mM ± 1.6 mM. The errors shown are the standard error from three separate measurements.

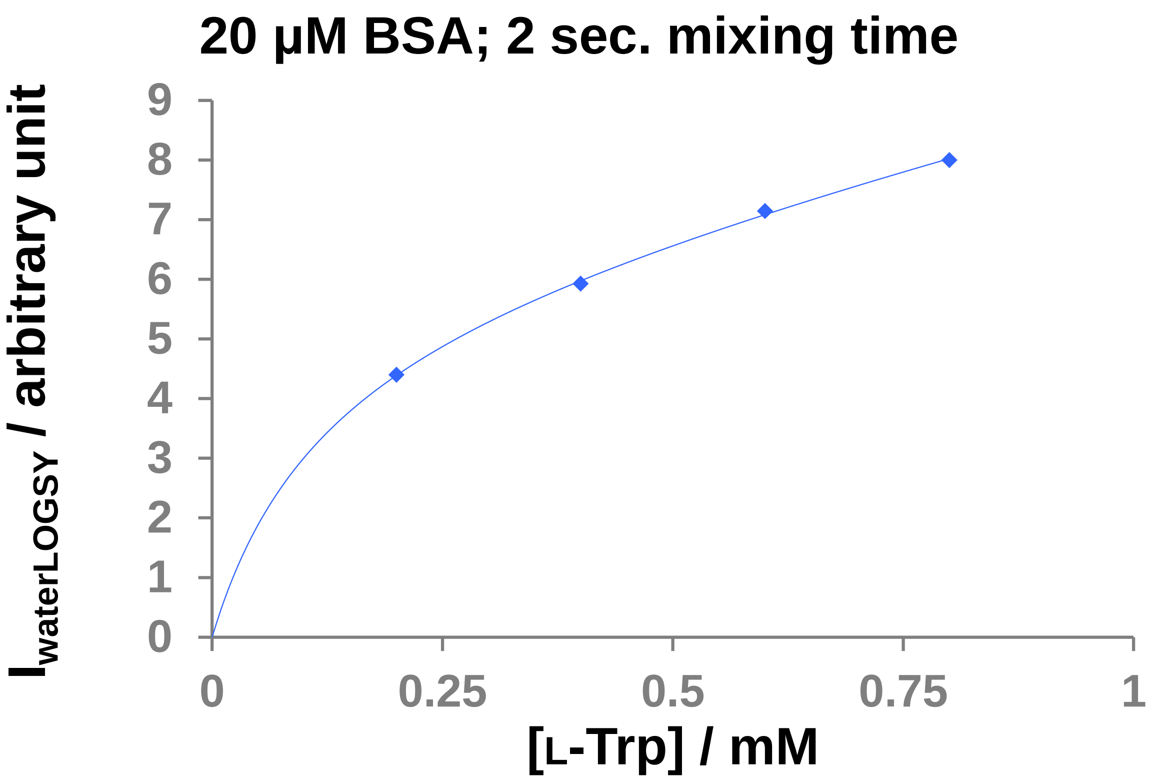

Supplementary Figure S8: Non-linear curve fitting to a corrected waterLOGSY binding isotherm. The mixing time was 2 seconds and the relaxation delay was 5 seconds. The system contained 20 μM bovine serum albumin (BSA) and 0.2–0.8 mM L-tryptophan (binder) in 50 mM Tris-D11 buffer (pH 7.5) at 90% H_2O and 10% D_2O and 0.02% NaN_3. The fitted K_D^{obs} was ~120 μM.

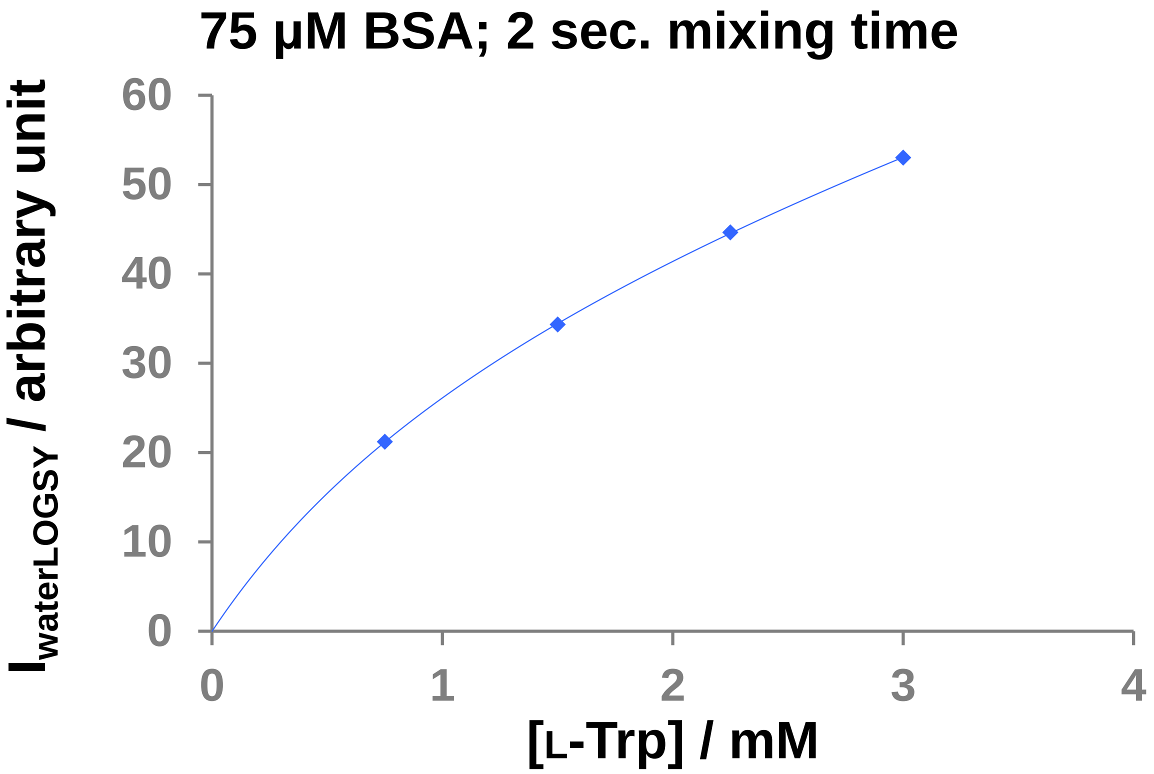

Supplementary Figure S9: Non-linear curve fitting to a corrected waterLOGSY binding isotherm. The mixing time was 2 seconds and the relaxation delay was 5 seconds. The system contained 75 µM bovine serum albumin (BSA) and 0.75–3.0 mM L-tryptophan (binder) in 50 mM Tris-D11 buffer (pH 7.5) at 90% H_2O and 10% D_2O and 0.02% NaN_3. The fitted K_D^{obs} was ~1.5 mM.

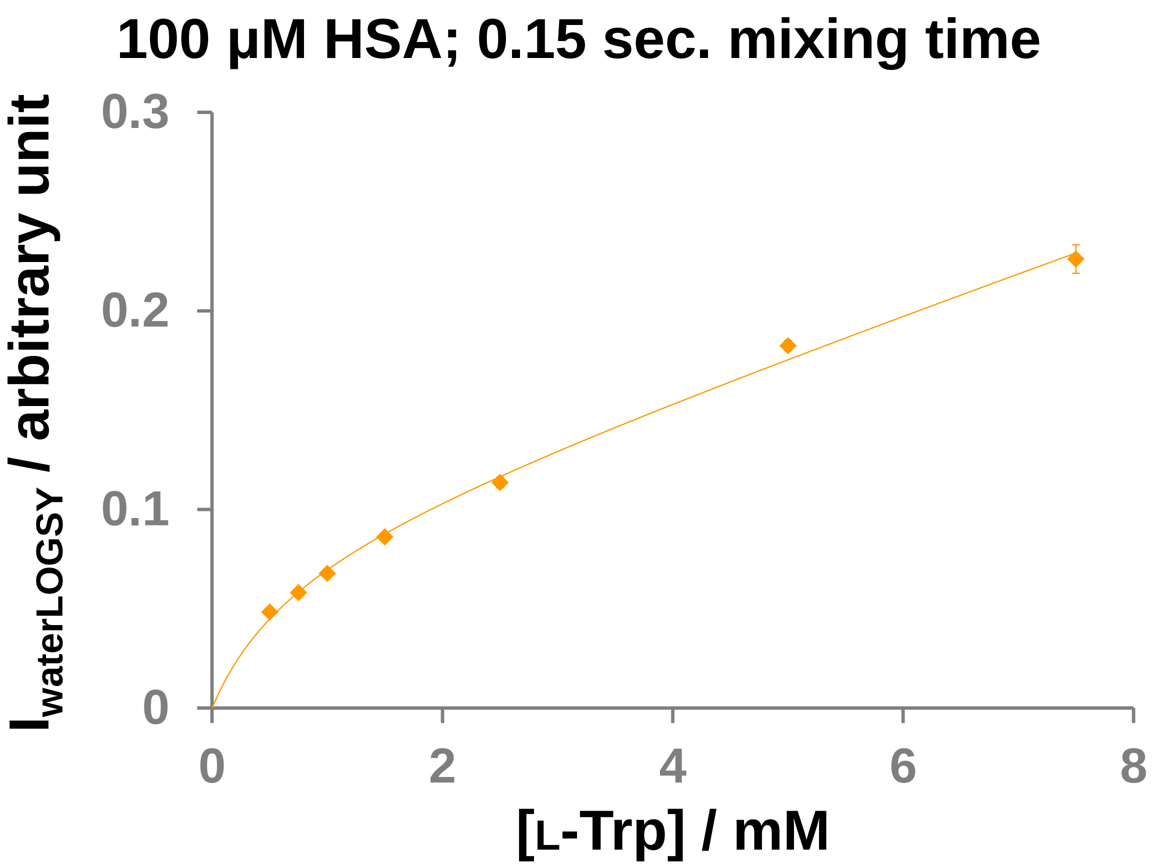

Supplementary Figure S10: Non-linear curve fitting to a corrected waterLOGSY binding isotherm. The mixing time was 0.15 seconds and the relaxation delay was 15 seconds. The system contained 100 µM human serum albumin (HSA) and 0.5–7.5 mM L-tryptophan (binder) in 50 mM sodium phosphate buffer (pH 7.5) at 90% H_2O and 10% D_2O. The fitted K_D^{obs} was 765 µM ± 140 µM. The errors shown are the standard error from three separate measurements.

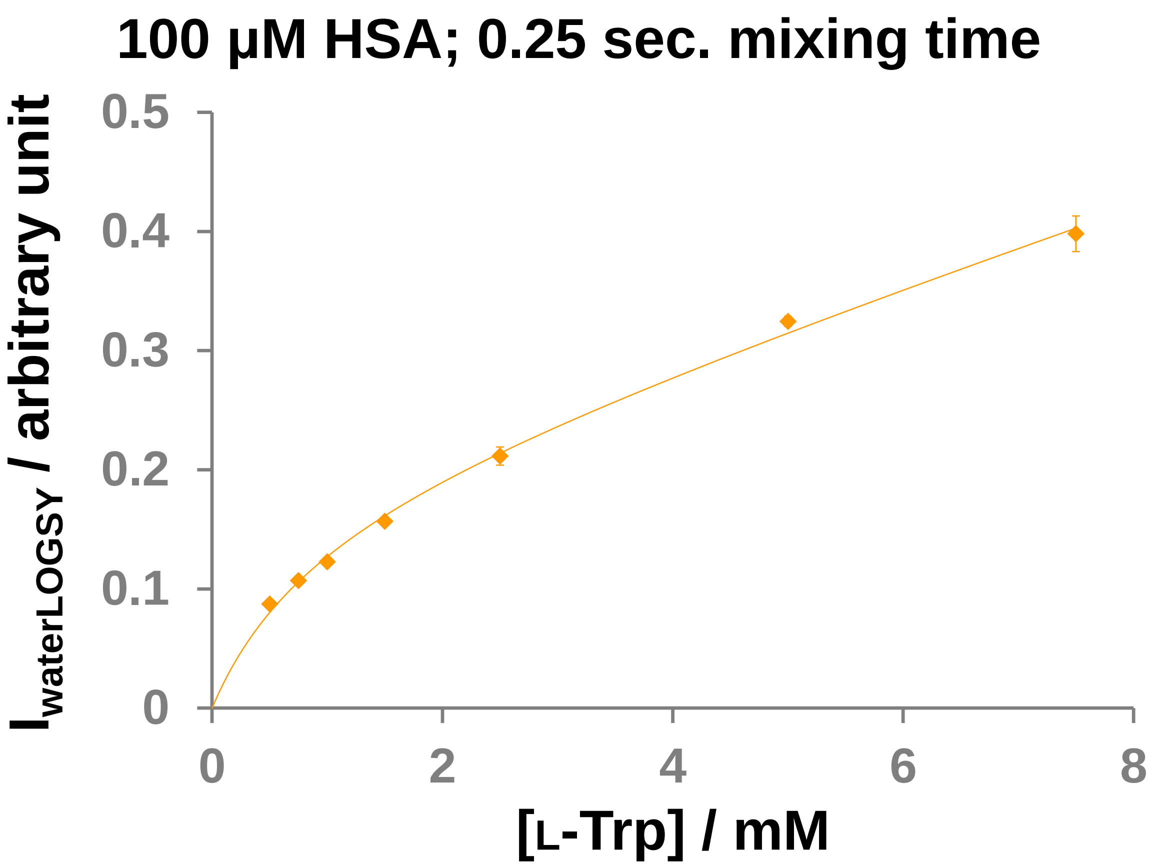

Supplementary Figure S11: Non-linear curve fitting to a corrected waterLOGSY binding isotherm. The mixing time was 0.25 seconds and the relaxation delay was 15 seconds. The system contained 100 µM human serum albumin (HSA) and 0.5–7.5 mM L-tryptophan (binder) in 50 mM sodium phosphate buffer (pH 7.5) at 90% H_2O and 10% D_2O. The fitted K_D^{obs} was 1.0 mM ± 0.2 mM. The errors shown are the standard error from three separate measurements.

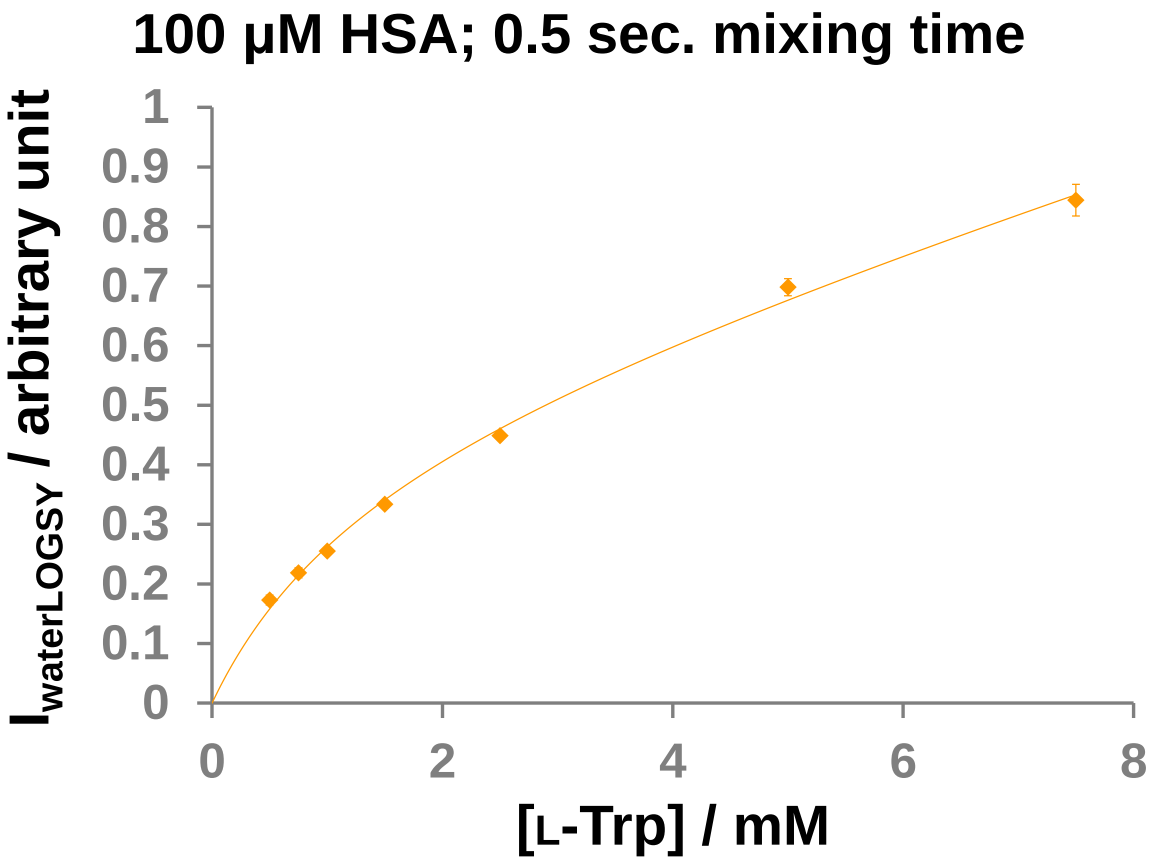

Supplementary Figure S12: Non-linear curve fitting to a corrected waterLOGSY binding isotherm. The mixing time was 0.5 seconds and the relaxation delay was 15 seconds. The system contained 100 µM human serum albumin (HSA) and 0.5–7.5 mM L-tryptophan (binder) in 50 mM sodium phosphate buffer (pH 7.5) at 90% H_2O and 10% D_2O. The fitted K_D^{obs} was 1.5 mM ± 0.4 mM. The errors shown are the standard error from three separate measurements.

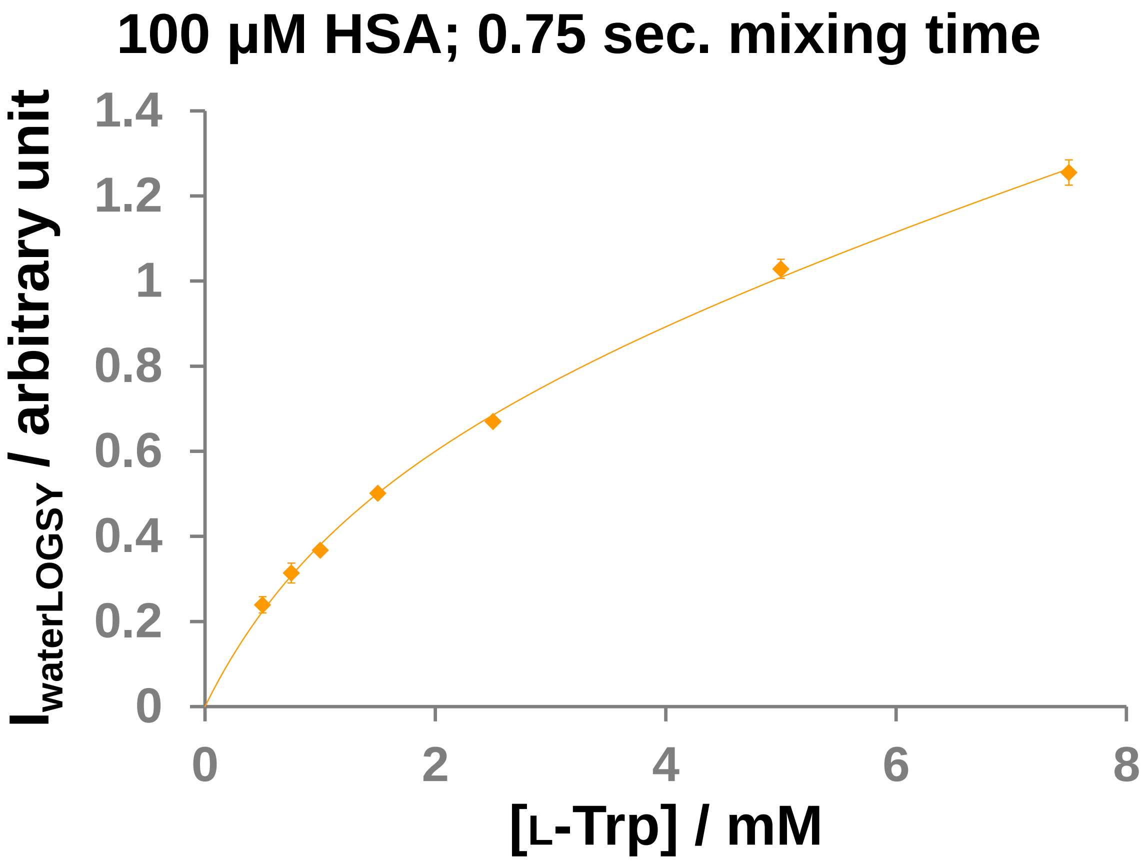

Supplementary Figure S13: Non-linear curve fitting to a corrected waterLOGSY binding isotherm. The mixing time was 0.75 seconds and the relaxation delay was 15 seconds. The system contained 100 µM human serum albumin (HSA) and 0.5–7.5 mM L-tryptophan (binder) in 50 mM sodium phosphate buffer (pH 7.5) at 90% H_2O and 10% D_2O. The fitted K_D^{obs} was 1.8 mM ± 0.1 mM. The errors shown are the standard error from three separate measurements.

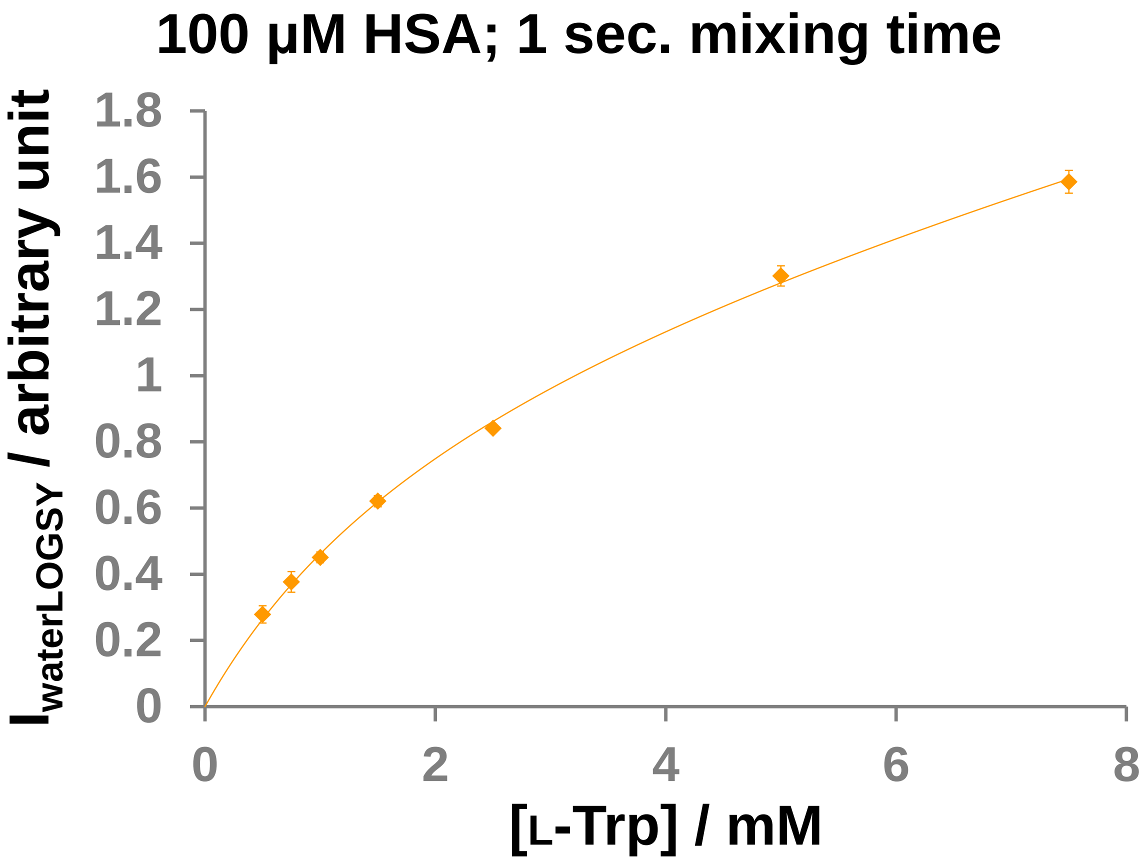

Supplementary Figure S14: Non-linear curve fitting to a corrected waterLOGSY binding isotherm. The mixing time was 1.0 seconds and the relaxation delay was 15 seconds. The system contained 100 µM human serum albumin (HSA) and 0.5–7.5 mM L-tryptophan (binder) in 50 mM sodium phosphate buffer (pH 7.5) at 90% H_2O and 10% D_2O. The fitted K_D^{obs} was 2.3 mM ± 0.2 mM. The errors shown are the standard error from three separate measurements.

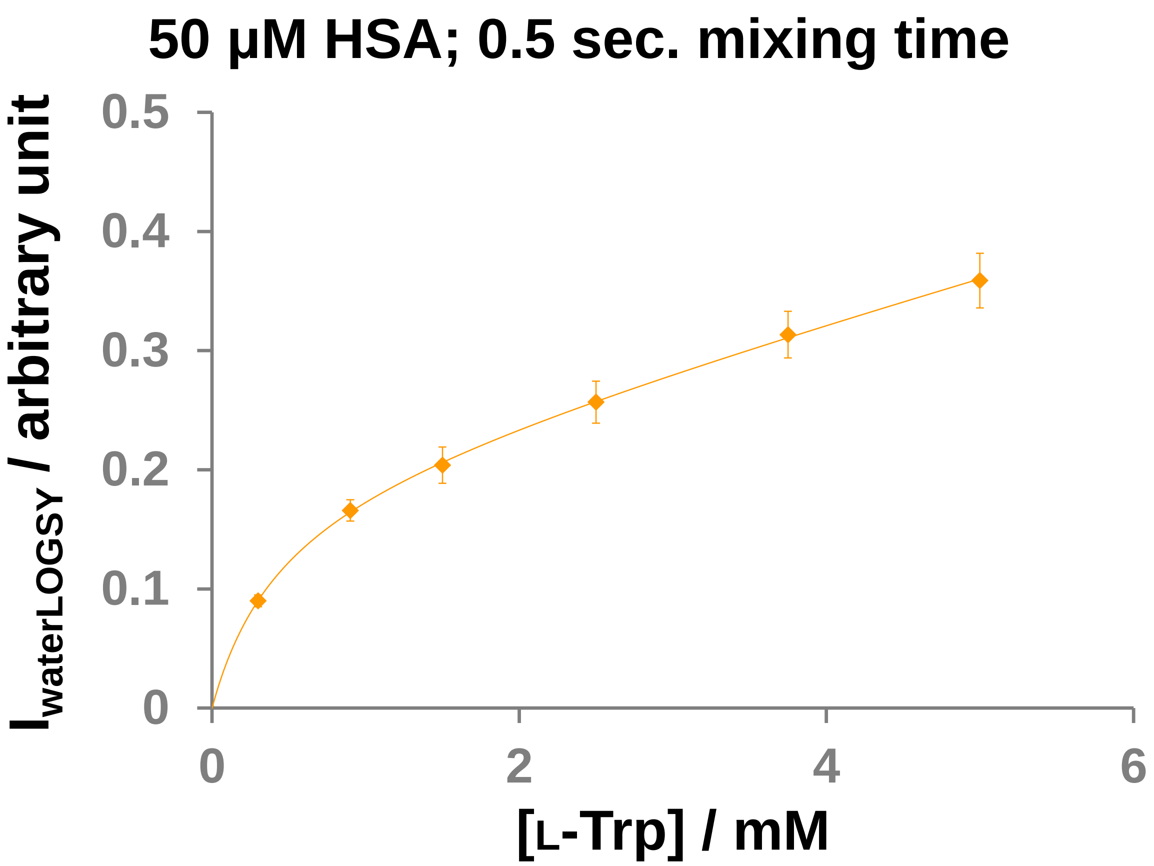

Supplementary Figure S15: Non-linear curve fitting to a corrected waterLOGSY binding isotherm. The mixing time was 0.5 seconds and the relaxation delay was 15 seconds. The system contained 50 µM human serum albumin (HSA) and 0.3–5.0 mM L-tryptophan (binder) in 50 mM sodium phosphate buffer (pH 7.5) at 90% H_2O and 10% D_2O. The fitted K_D^{obs} was 475 µM ± 165 µM. The errors shown are the standard error from three separate measurements.

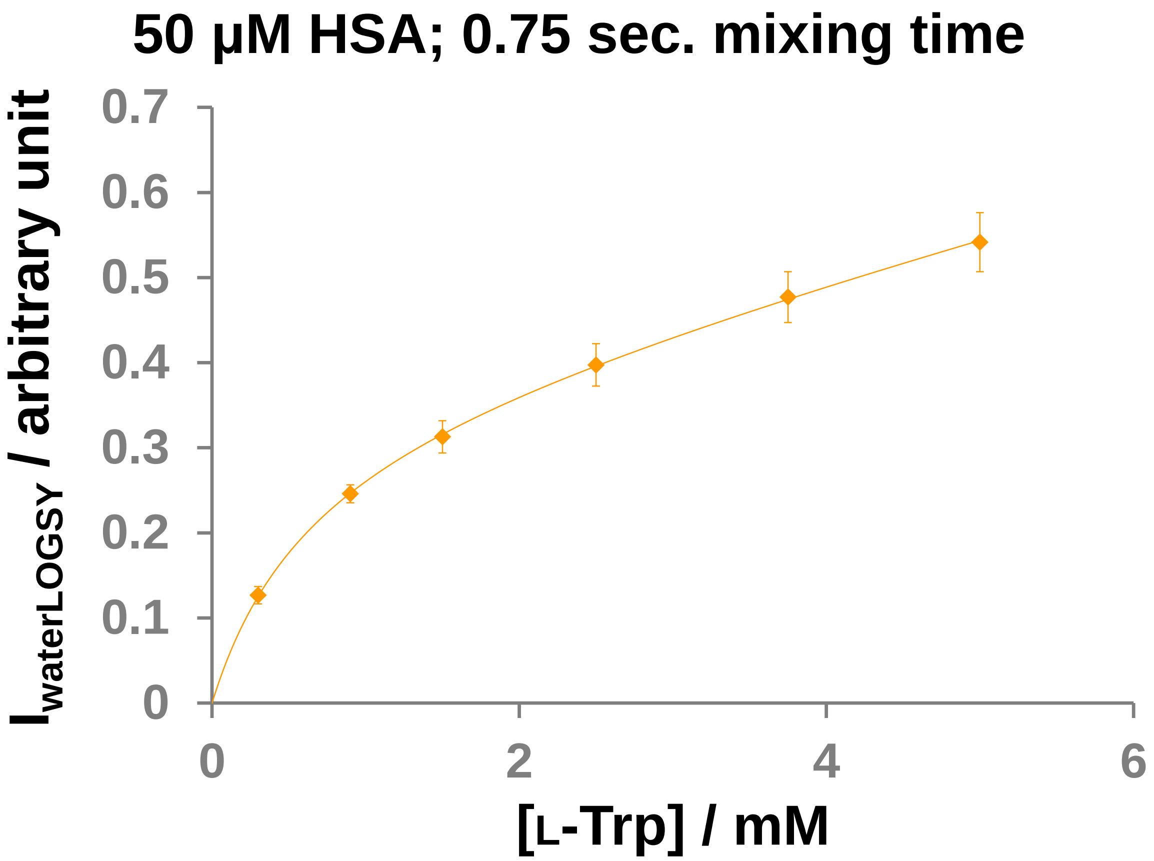

Supplementary Figure S16: Non-linear curve fitting to a corrected waterLOGSY binding isotherm. The mixing time was 0.75 seconds and the relaxation delay was 15 seconds. The system contained 50 µM human serum albumin (HSA) and 0.3–5.0 mM L-tryptophan (binder) in 50 mM sodium phosphate buffer (pH 7.5) at 90% H_2O and 10% D_2O. The fitted K_D^{obs} was 715 µM ± 230 µM. The errors shown are the standard error from three separate measurements.

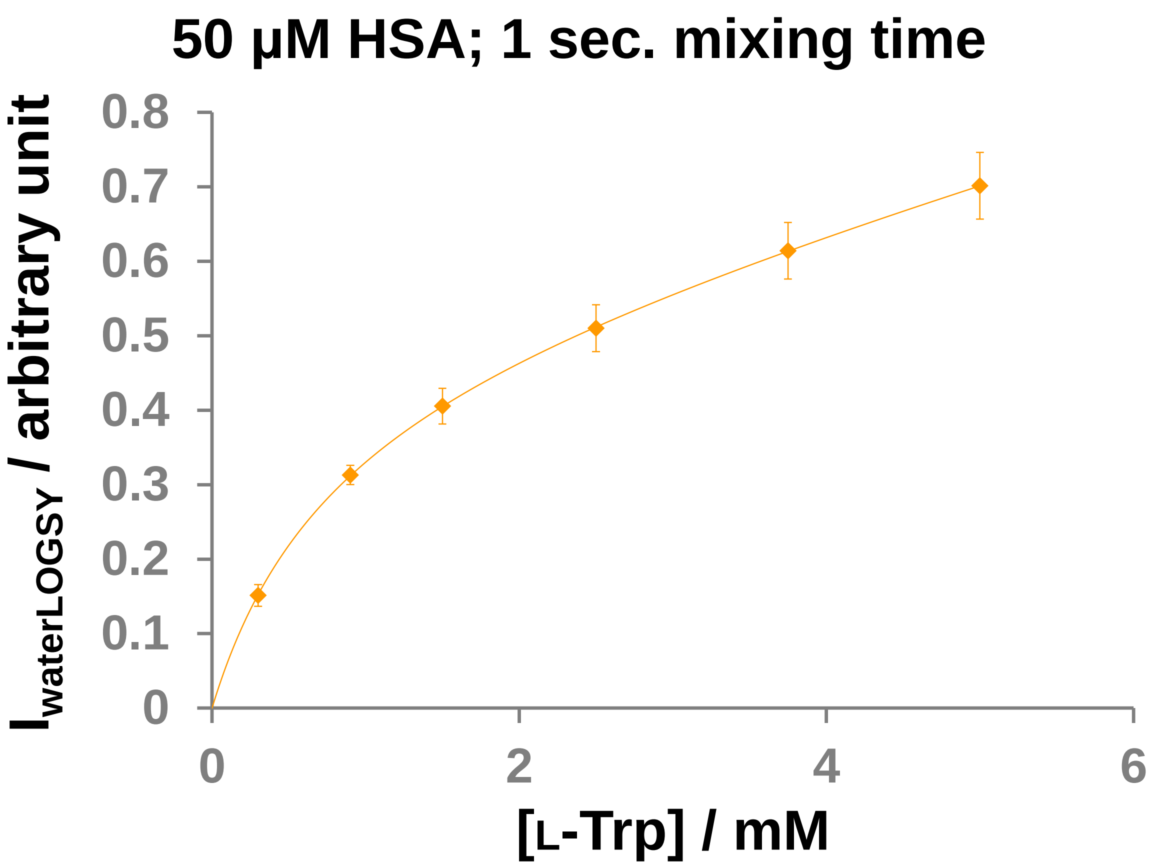

Supplementary Figure S17: Non-linear curve fitting to a corrected waterLOGSY binding isotherm. The mixing time was 1.0 seconds and the relaxation delay was 15 seconds. The system contained 50 µM human serum albumin (HSA) and 0.3–5.0 mM L-tryptophan (binder) in 50 mM sodium phosphate buffer (pH 7.5) at 90% H_2O and 10% D_2O. The fitted K_D^{obs} was 845 µM ± 270 µM. The errors shown are the standard error from three separate measurements.

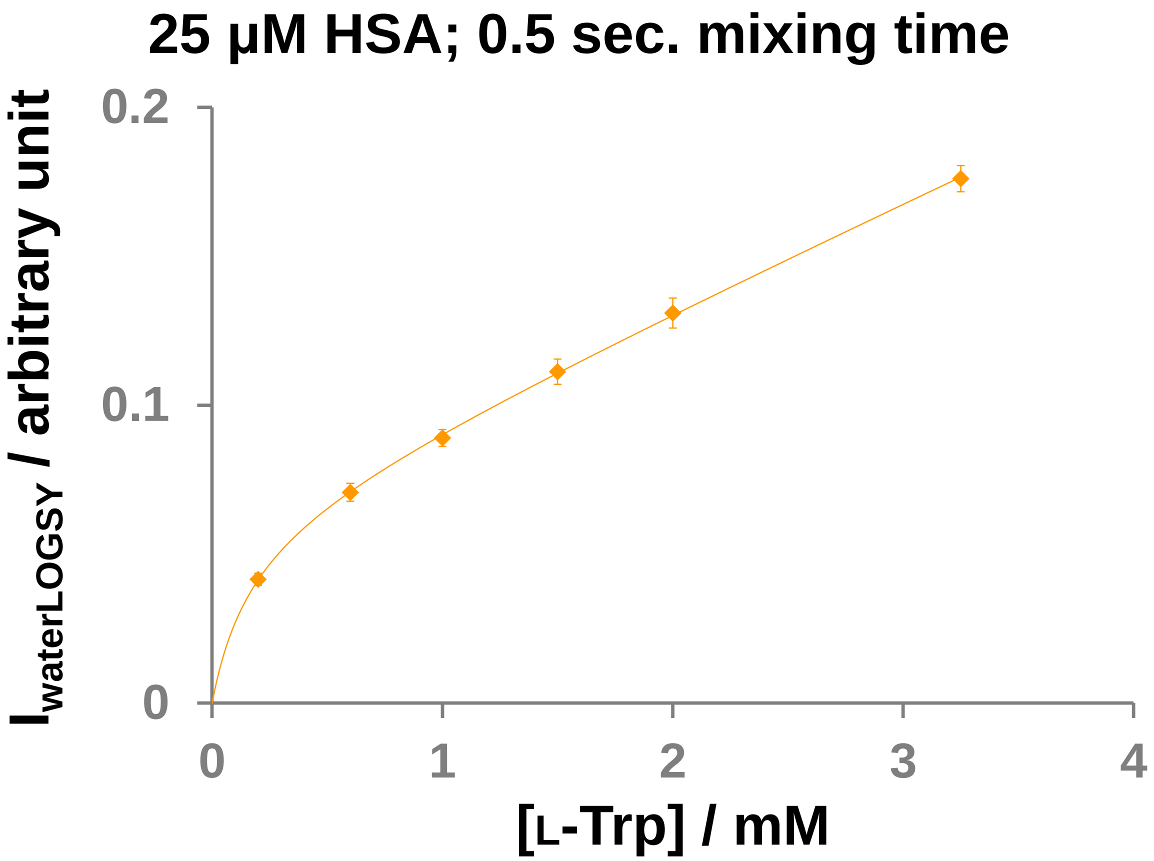

Supplementary Figure S18: Non-linear curve fitting to a corrected waterLOGSY binding isotherm. The mixing time was 0.5 seconds and the relaxation delay was 15 seconds. The system contained 25 µM human serum albumin (HSA) and 0.2–3.25 mM L-tryptophan (binder) in 50 mM sodium phosphate buffer (pH 7.5) at 90% H_2O and 10% D_2O. The fitted K_D^{obs} was 185 µM ± 50 µM. The errors shown are the standard error from three separate measurements.

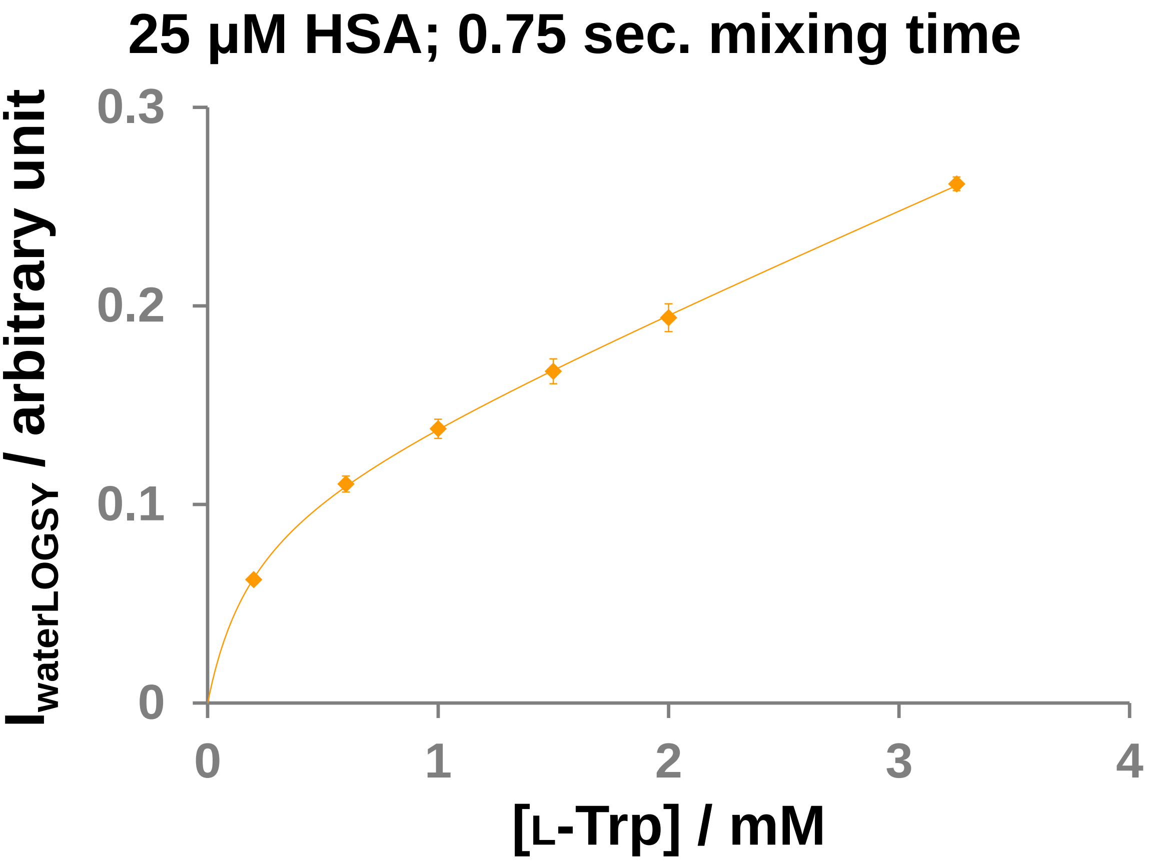

Supplementary Figure S19: Non-linear curve fitting to a corrected waterLOGSY binding isotherm. The mixing time was 0.75 seconds and the relaxation delay was 15 seconds. The system contained 25 µM human serum albumin (HSA) and 0.2–3.25 mM L-tryptophan (binder) in 50 mM sodium phosphate buffer (pH 7.5) at 90% H_2O and 10% D_2O. The fitted K_D^{obs} was 200 µM ± 45 µM. The errors shown are the standard error from three separate measurements.

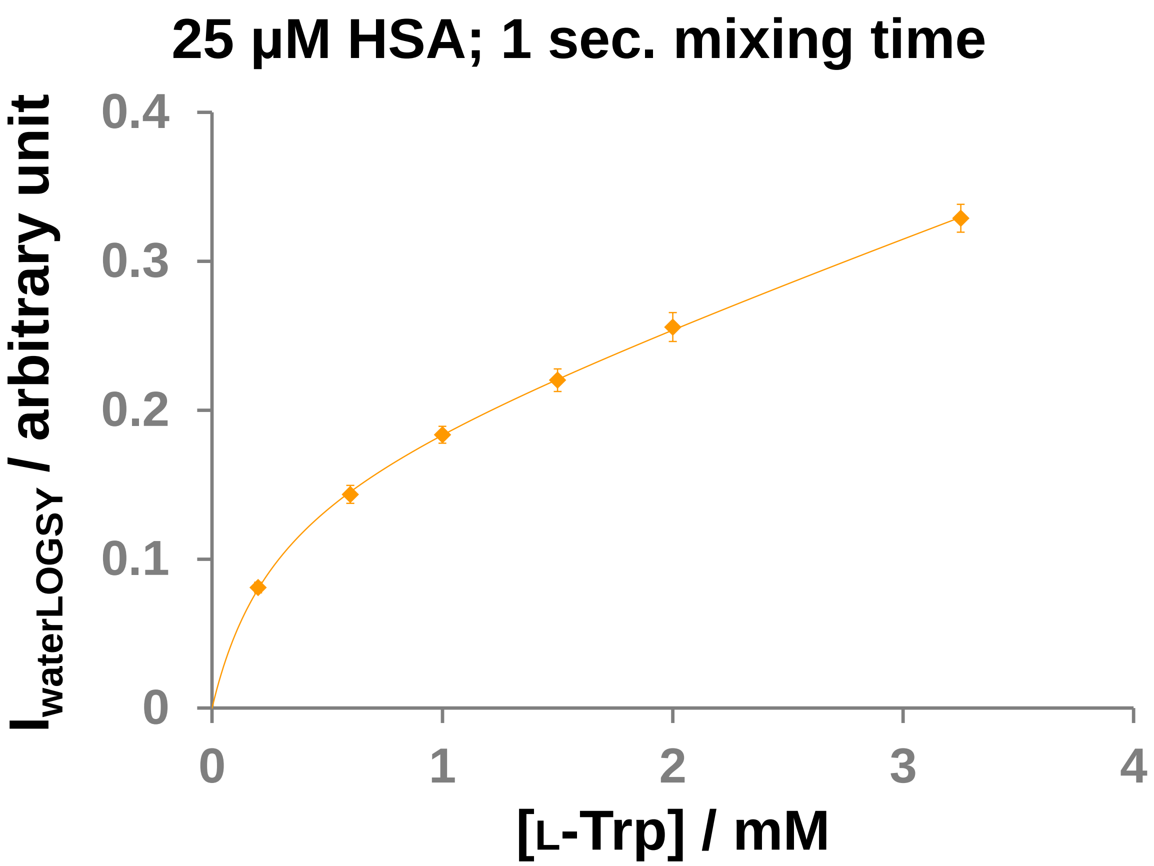

Supplementary Figure S20: Non-linear curve fitting to a corrected waterLOGSY binding isotherm. The mixing time was 1.0 seconds and the relaxation delay was 15 seconds. The system contained 25 µM human serum albumin (HSA) and 0.2–3.25 mM L-tryptophan (binder) in 50 mM sodium phosphate buffer (pH 7.5) at 90% H_2O and 10% D_2O. The fitted K_D^{obs} was 280 µM ± 40 µM. The errors shown are the standard error from three separate measurements.

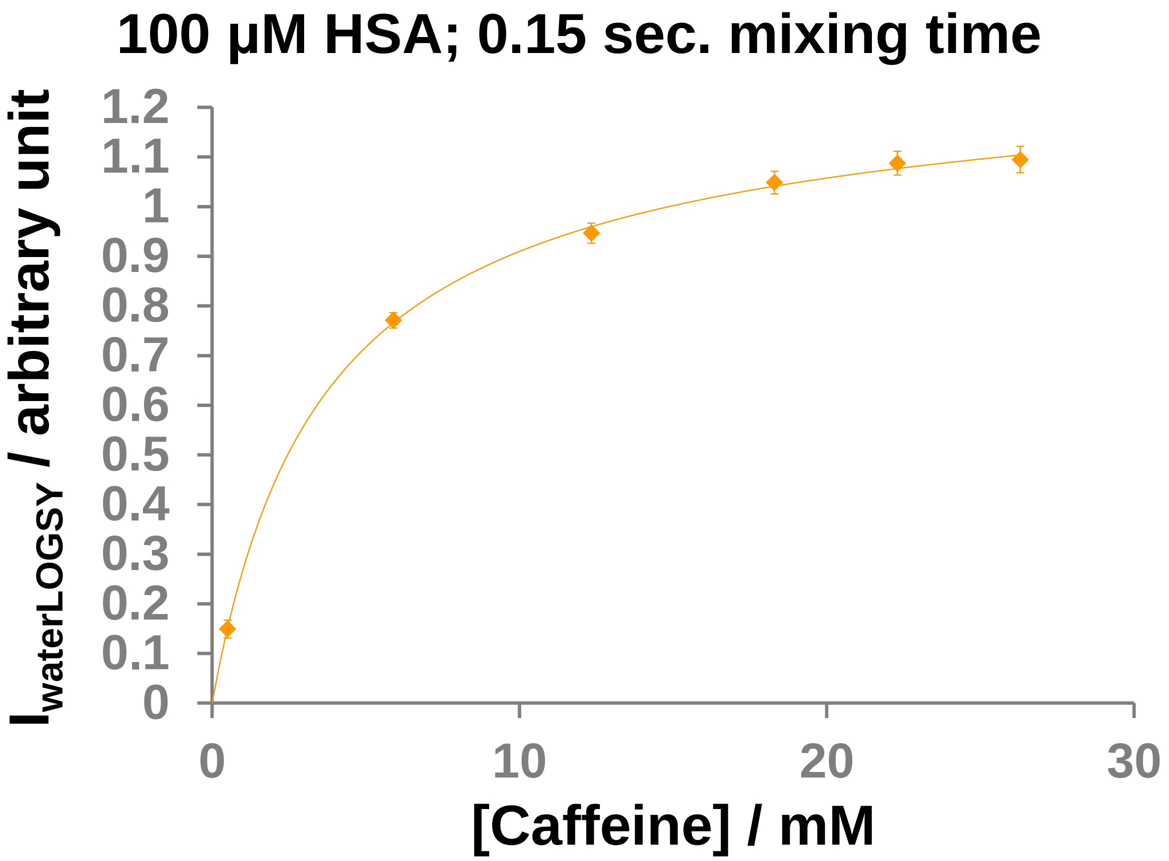

Supplementary Figure S21: Non-linear curve fitting to a corrected waterLOGSY binding isotherm. The mixing time was 0.15 seconds and the relaxation delay was 15 seconds. The system contained 25 μM human serum albumin (HSA) and 0.5–26.3 mM caffeine (binder) in 50 mM sodium phosphate buffer (pH 7.5) at 90% H_2O and 10% D_2O. The fitted K_D^{obs} was 3.2 mM ± 0.3 mM. The errors shown are the standard error from three separate measurements.

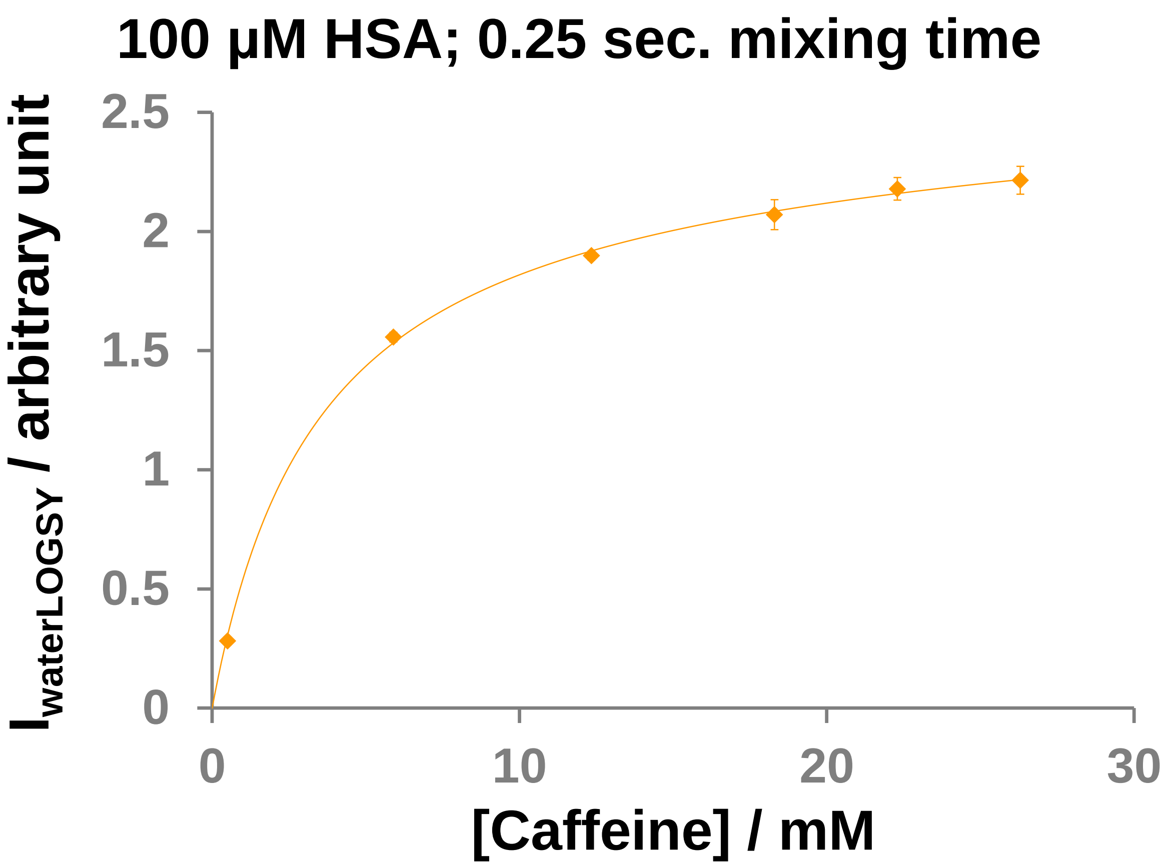

Supplementary Figure S22: Non-linear curve fitting to a corrected waterLOGSY binding isotherm. The mixing time was 0.25 seconds and the relaxation delay was 15 seconds. The system contained 25 μM human serum albumin (HSA) and 0.5–26.3 mM caffeine (binder) in 50 mM sodium phosphate buffer (pH 7.5) at 90% H_2O and 10% D_2O. The fitted K_D^{obs} was 3.3 mM ± 0.3 mM. The errors shown are the standard error from three separate measurements.

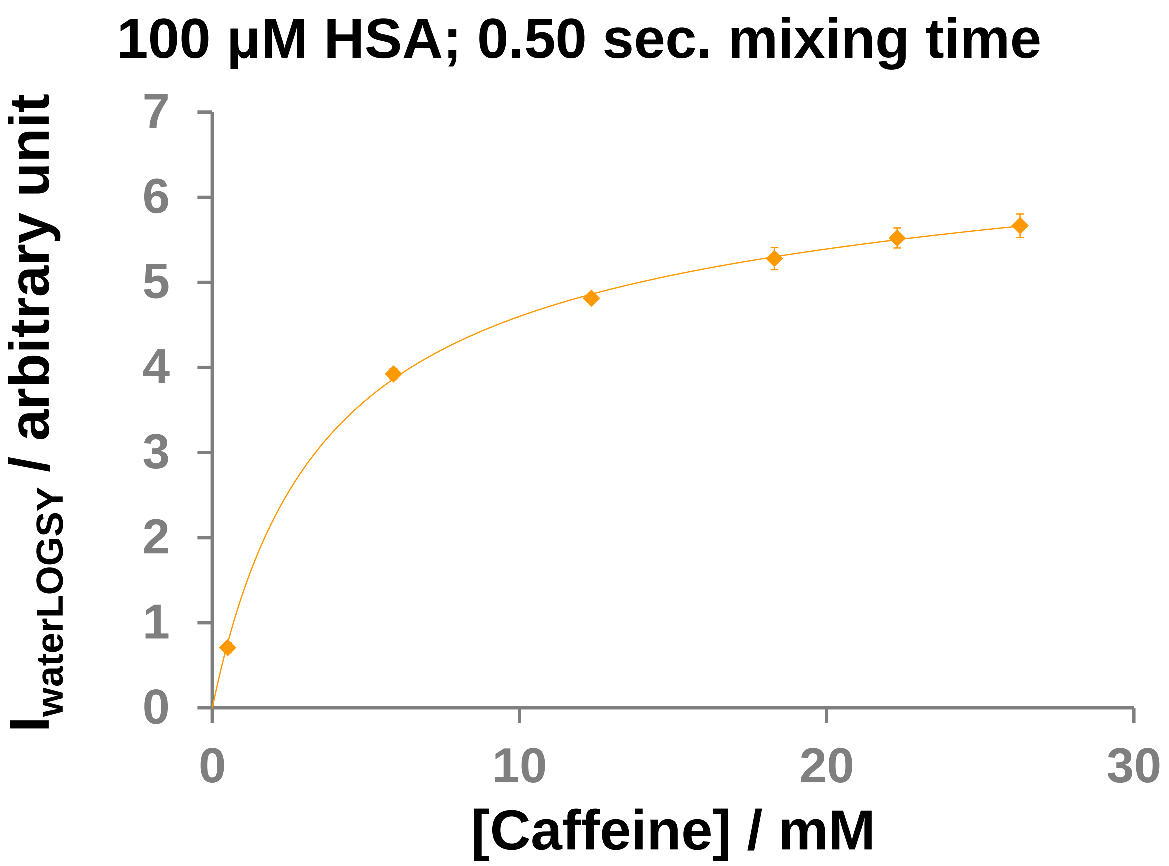

Supplementary Figure S23: Non-linear curve fitting to a corrected waterLOGSY binding isotherm. The mixing time was 0.50 seconds and the relaxation delay was 15 seconds. The system contained 100 µM human serum albumin (HSA) and 0.5–26.3 mM caffeine (binder) in 50 mM sodium phosphate buffer (pH 7.5) at 90% H_2O and 10% D_2O. The fitted K_D^{obs} was 3.4 mM ± 0.3 mM. The errors shown are the standard error from three separate measurements.

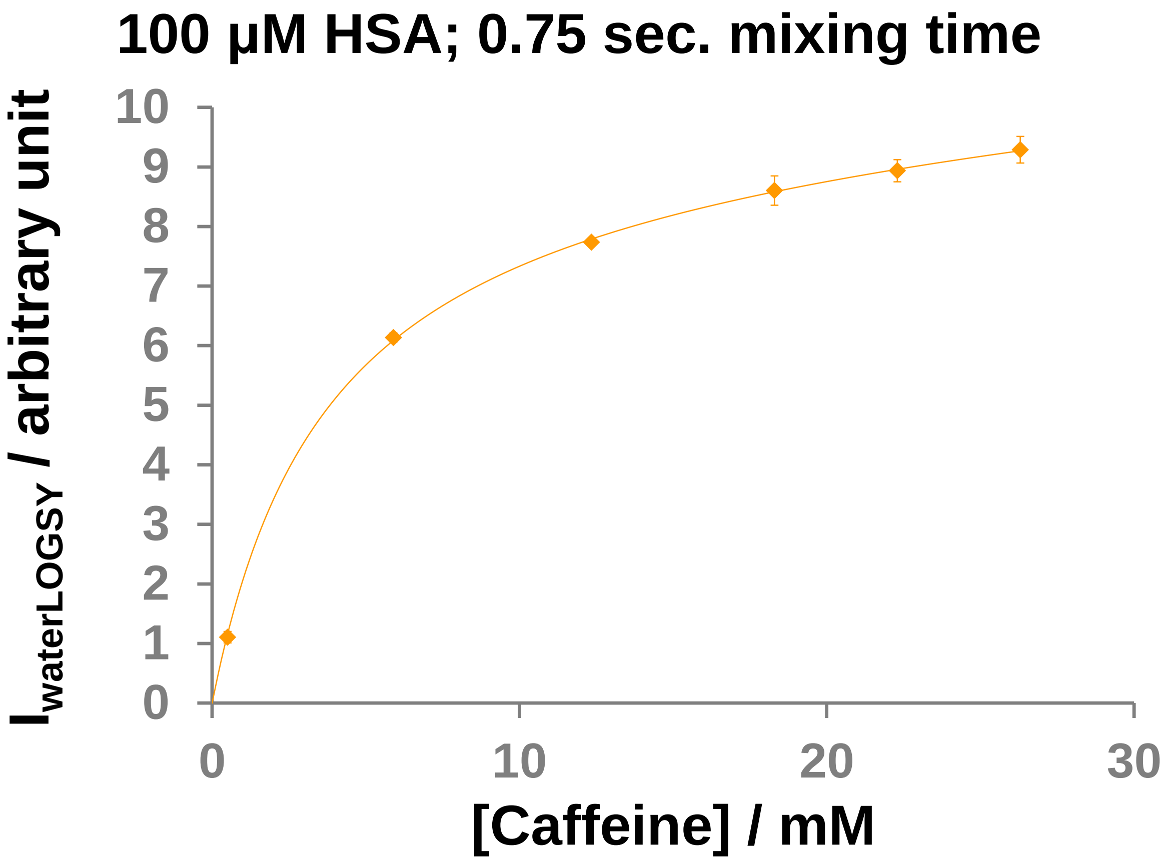

Supplementary Figure S24: Non-linear curve fitting to a corrected waterLOGSY binding isotherm. The mixing time was 0.75 seconds and the relaxation delay was 15 seconds. The system contained 100 µM human serum albumin (HSA) and 0.5–26.3 mM caffeine (binder) in 50 mM sodium phosphate buffer (pH 7.5) at 90% H_2O and 10% D_2O. The fitted K_D^{obs} was 3.7 mM ± 0.4 mM. The errors shown are the standard error from three separate measurements.

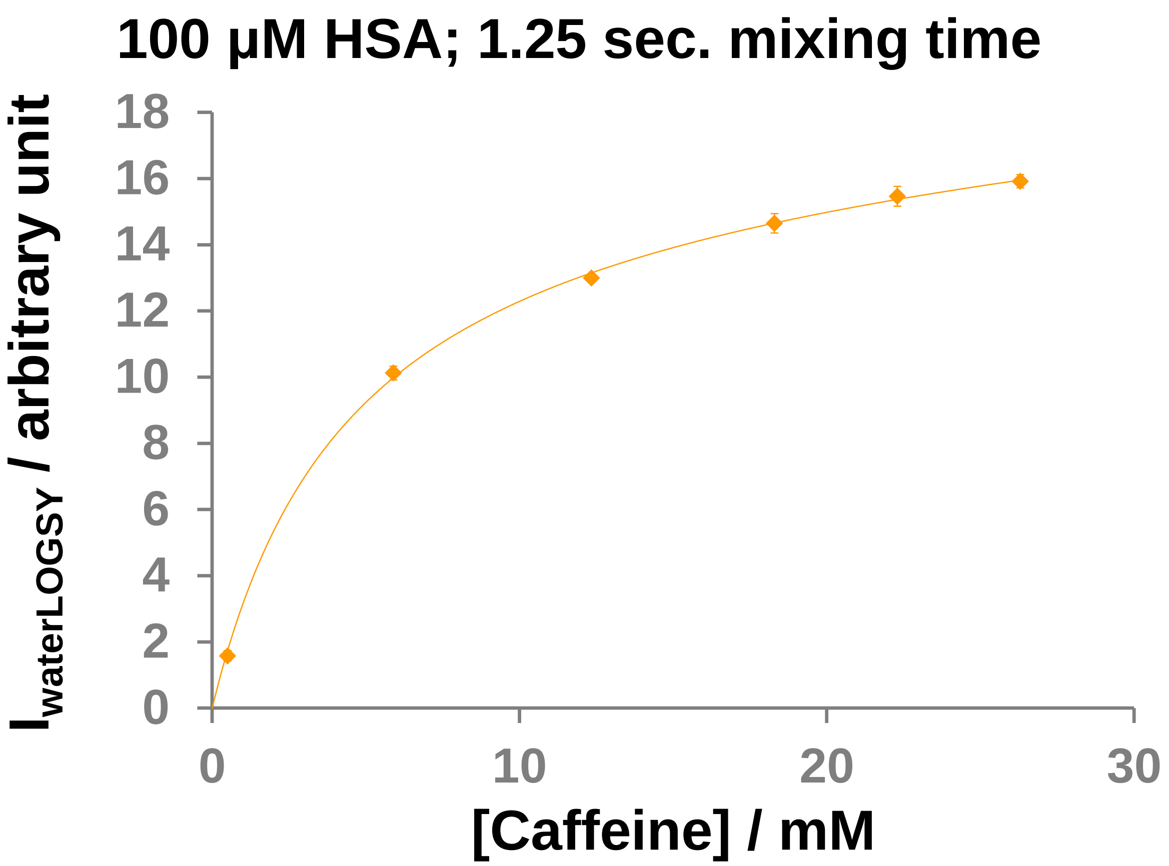

Supplementary Figure S25: Non-linear curve fitting to a corrected waterLOGSY binding isotherm. The mixing time was 1.25 seconds and the relaxation delay was 15 seconds. The system contained 100 μM human serum albumin (HSA) and 0.5–26.3 mM caffeine (binder) in 50 mM sodium phosphate buffer (pH 7.5) at 90% H_2O and 10% D_2O. The fitted K_D^{obs} was 4.5 mM ± 0.3 mM. The errors shown are the standard error from three separate measurements.

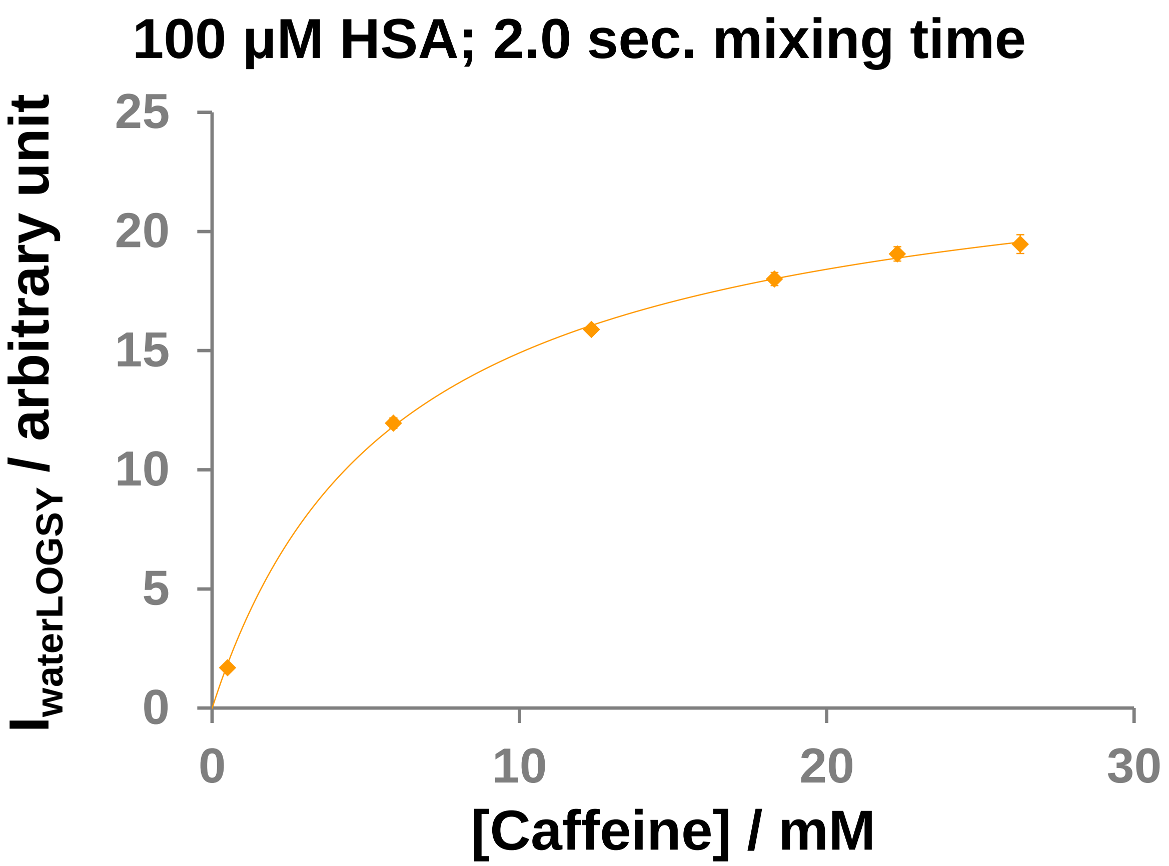

Supplementary Figure S26: Non-linear curve fitting to a corrected waterLOGSY binding isotherm. The mixing time was 2.0 seconds and the relaxation delay was 15 seconds. The system contained 100 µM human serum albumin (HSA) and 0.5–26.3 mM caffeine (binder) in 50 mM sodium phosphate buffer (pH 7.5) at 90% H_2O and 10% D_2O. The fitted K_D^{obs} was 5.5 mM ± 0.4 mM. The errors shown are the standard error from three separate measurements.

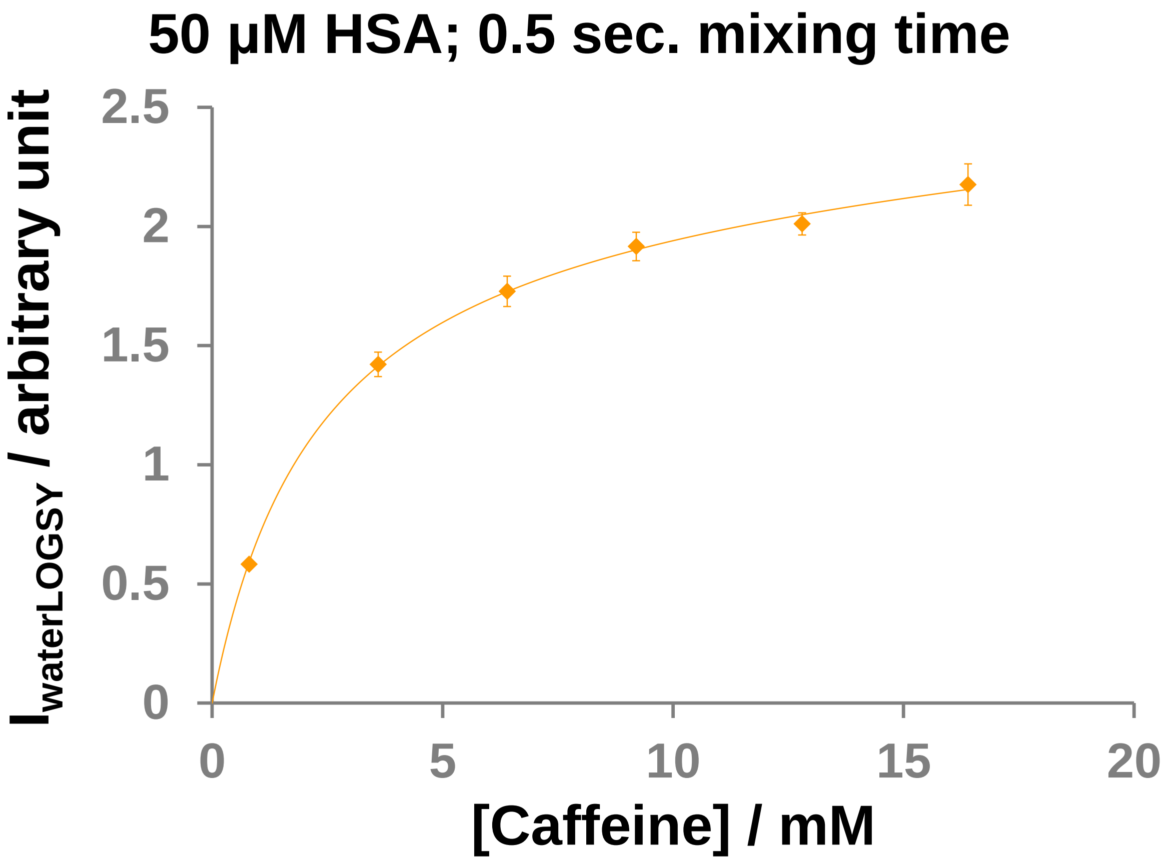

Supplementary Figure S27: Non-linear curve fitting to a corrected waterLOGSY binding isotherm. The mixing time was 0.5 seconds and the relaxation delay was 15 seconds. The system contained 50 µM human serum albumin (HSA) and 0.8–16.4 mM caffeine (binder) in 50 mM sodium phosphate buffer (pH 7.5) at 90% H_2O and 10% D_2O. The fitted K_D^{obs} was 2.3 mM ± 0.1 mM. The errors shown are the standard error from three separate measurements.

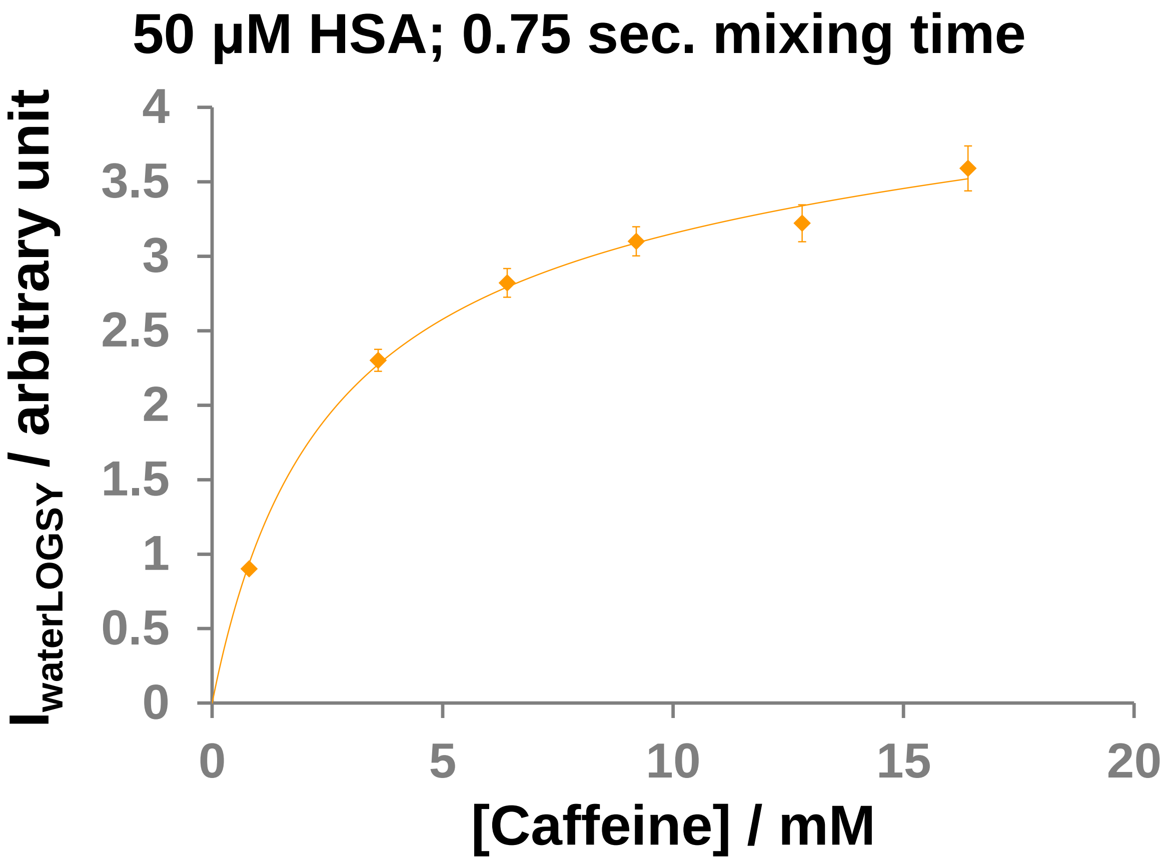

Supplementary Figure S28: Non-linear curve fitting to a corrected waterLOGSY binding isotherm. The mixing time was 0.75 seconds and the relaxation delay was 15 seconds. The system contained 50 µM human serum albumin (HSA) and 0.8–16.4 mM caffeine (binder) in 50 mM sodium phosphate buffer (pH 7.5) at 90% H_2O and 10% D_2O. The fitted K_D^{obs} was 2.4 mM ± 0.1 mM. The errors shown are the standard error from three separate measurements.

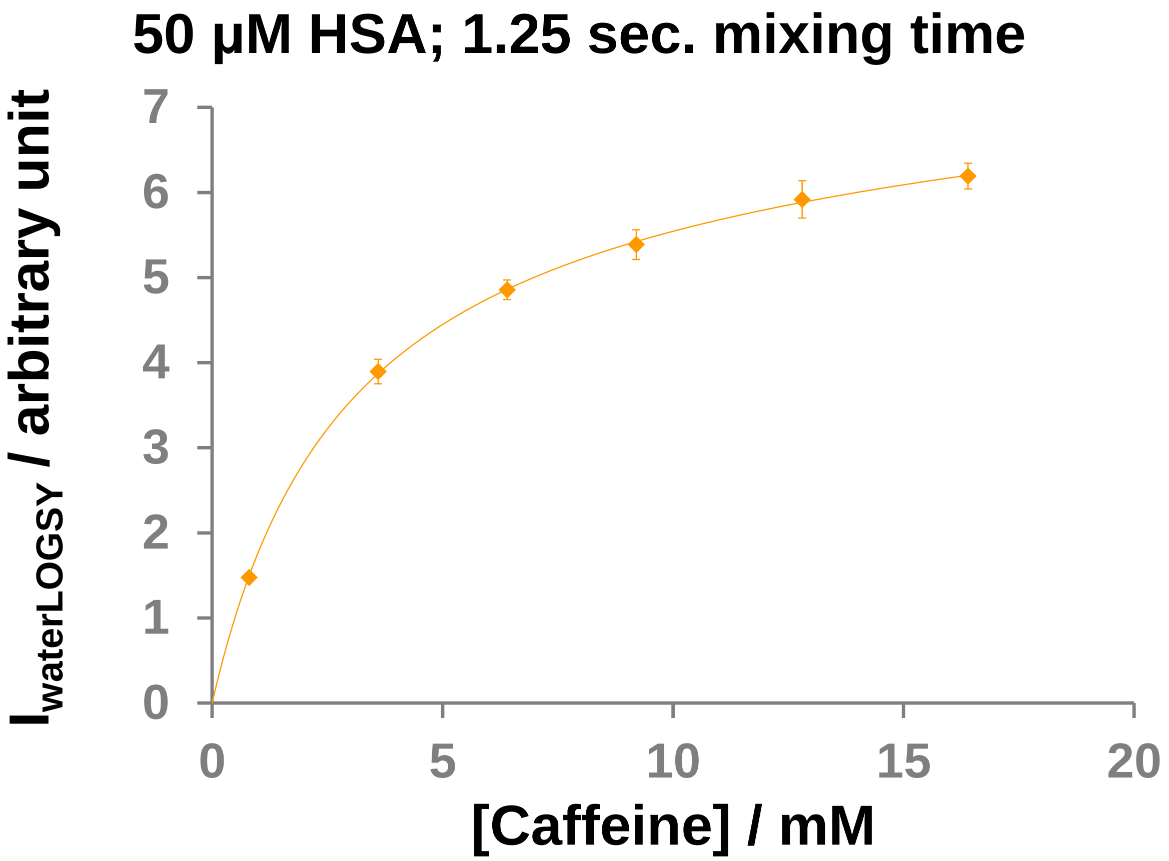

Supplementary Figure S29: Non-linear curve fitting to a corrected waterLOGSY binding isotherm. The mixing time was 1.25 seconds and the relaxation delay was 15 seconds. The system contained 50 µM human serum albumin (HSA) and 0.8–16.4 mM caffeine (binder) in 50 mM sodium phosphate buffer (pH 7.5) at 90% H_2O and 10% D_2O. The fitted K_D^{obs} was 2.9 mM ± 0.2 mM. The errors shown are the standard error from three separate measurements.

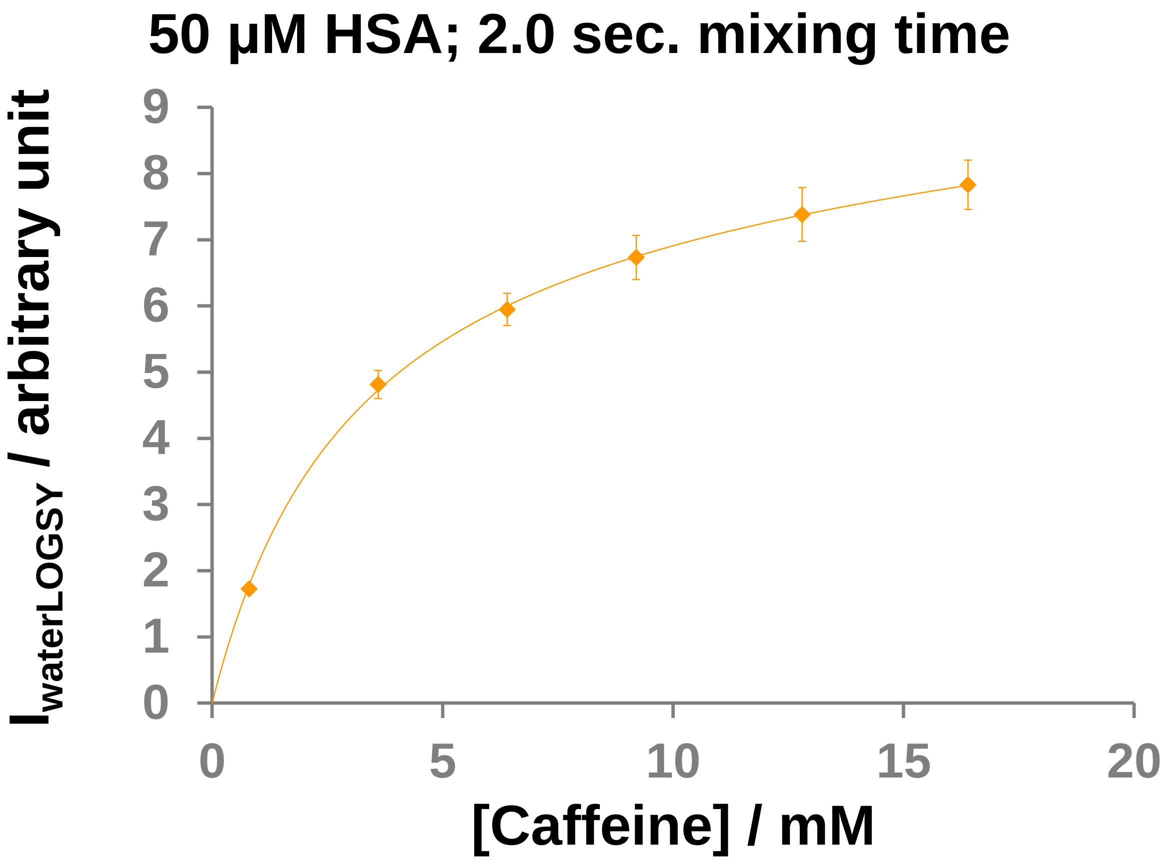

Supplementary Figure S30: Non-linear curve fitting to a corrected waterLOGSY binding isotherm. The mixing time was 2.0 seconds and the relaxation delay was 15 seconds. The system contained 50 µM human serum albumin (HSA) and 0.8–16.4 mM caffeine (binder) in 50 mM sodium phosphate buffer (pH 7.5) at 90% H_2O and 10% D_2O. The fitted K_D^{obs} was 3.1 mM ± 0.3 mM. The errors shown are the standard error from three separate measurements.

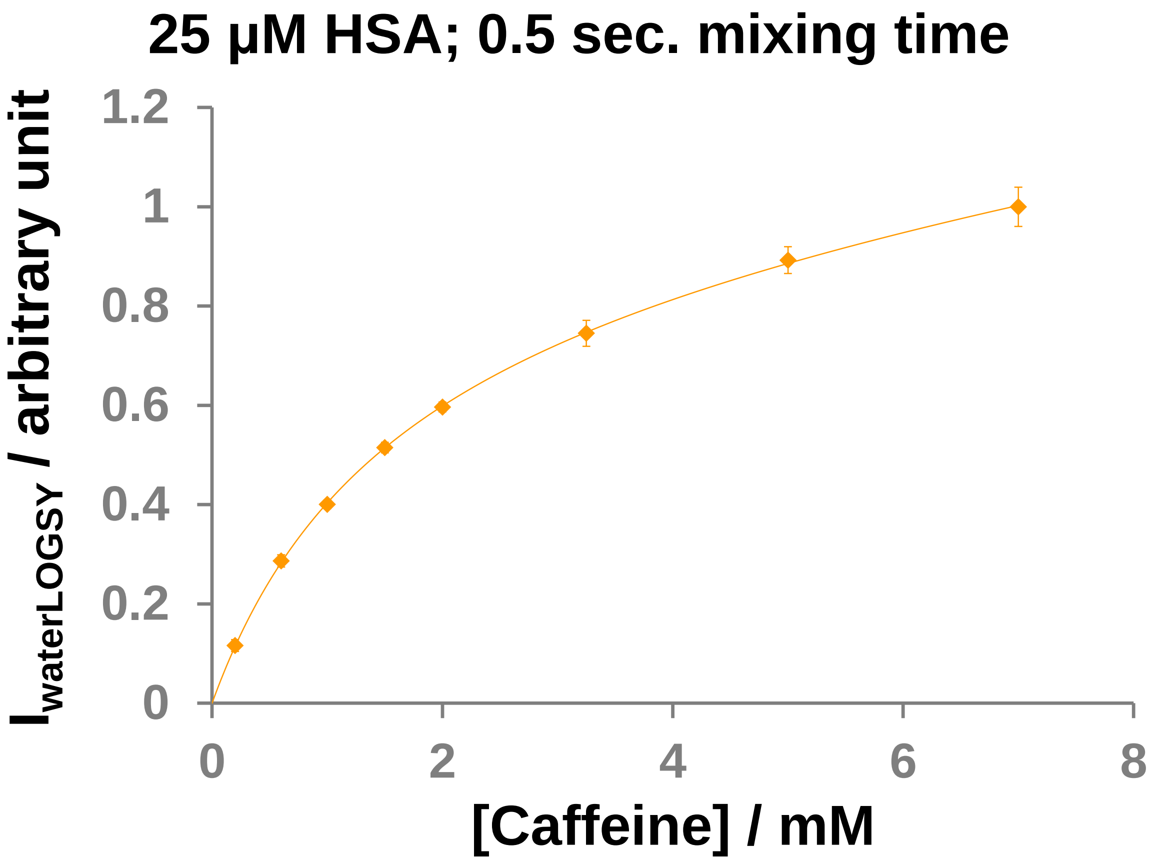

Supplementary Figure S31: Non-linear curve fitting to a corrected waterLOGSY binding isotherm. The mixing time was 0.5 seconds and the relaxation delay was 15 seconds. The system contained 25 µM human serum albumin (HSA) and 0.2–7.0 mM caffeine (binder) in 50 mM sodium phosphate buffer (pH 7.5) at 90% H_2O and 10% D_2O. The fitted K_D^{obs} was 1.6 mM ± 0.1 mM. The errors shown are the standard error from three separate measurements.

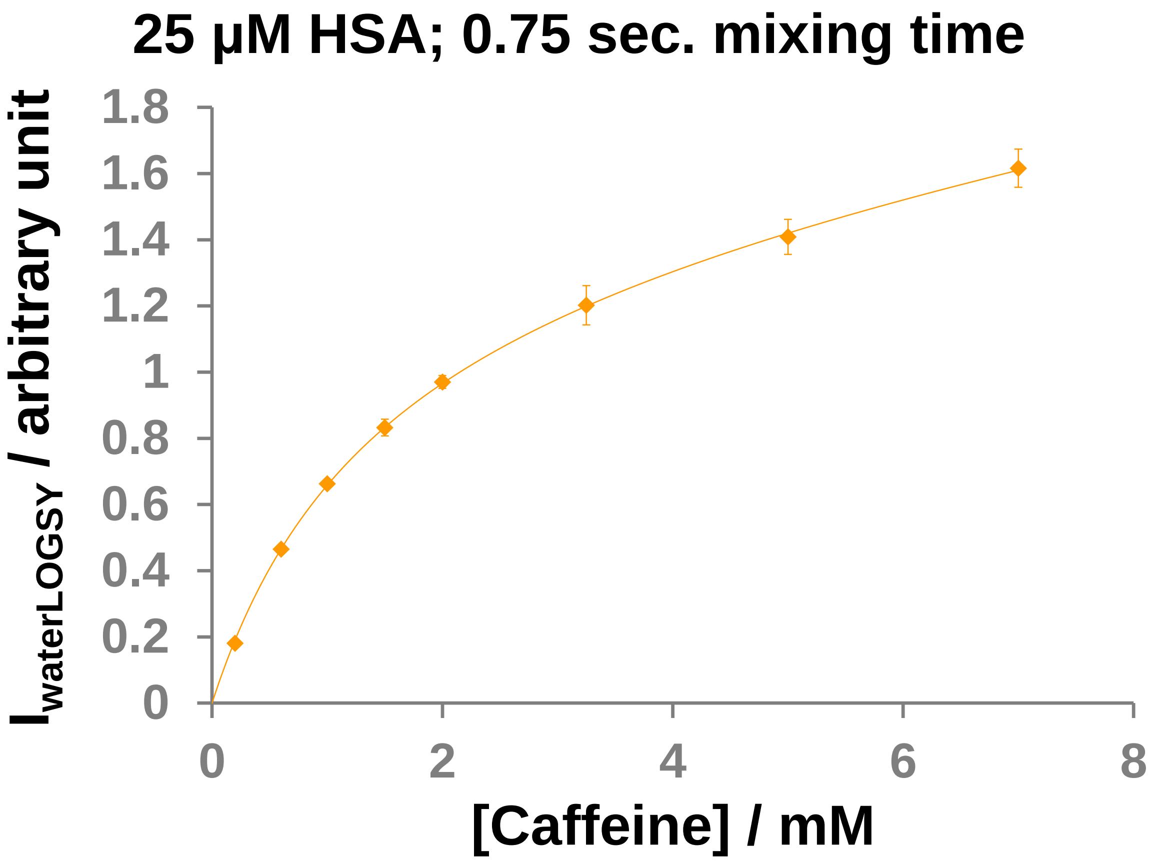

Supplementary Figure S32: Non-linear curve fitting to a corrected waterLOGSY binding isotherm. The mixing time was 0.75 seconds and the relaxation delay was 15 seconds. The system contained 25 µM human serum albumin (HSA) and 0.2–7.0 mM caffeine (binder) in 50 mM sodium phosphate buffer (pH 7.5) at 90% H_2O and 10% D_2O. The fitted K_D^{obs} was 1.5 mM ± 0.3 mM. The errors shown are the standard error from three separate measurements.

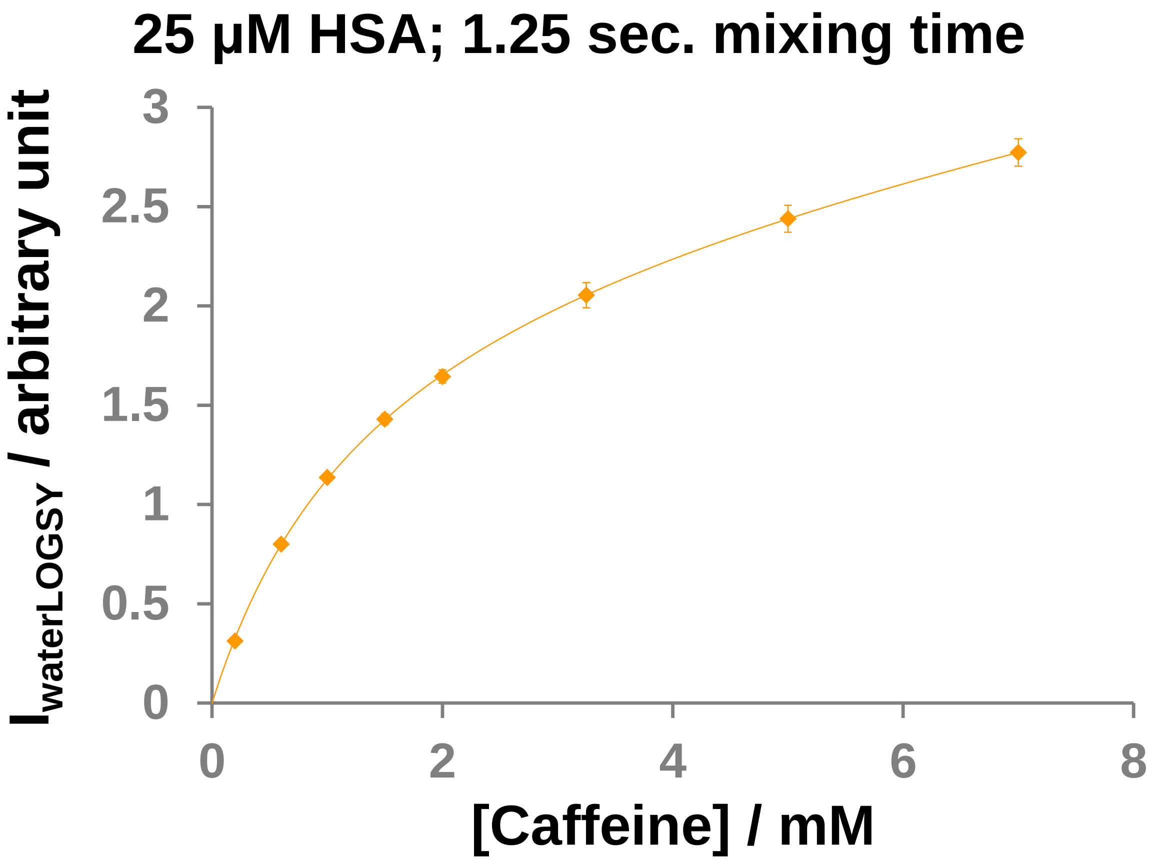

Supplementary Figure S33: Non-linear curve fitting to a corrected waterLOGSY binding isotherm. The mixing time was 1.25 seconds and the relaxation delay was 15 seconds. The system contained 25 µM human serum albumin (HSA) and 0.2–7.0 mM caffeine (binder) in 50 mM sodium phosphate buffer (pH 7.5) at 90% H_2O and 10% D_2O. The fitted K_D^{obs} was 1.4 mM ± 0.2 mM. The errors shown are the standard error from three separate measurements.

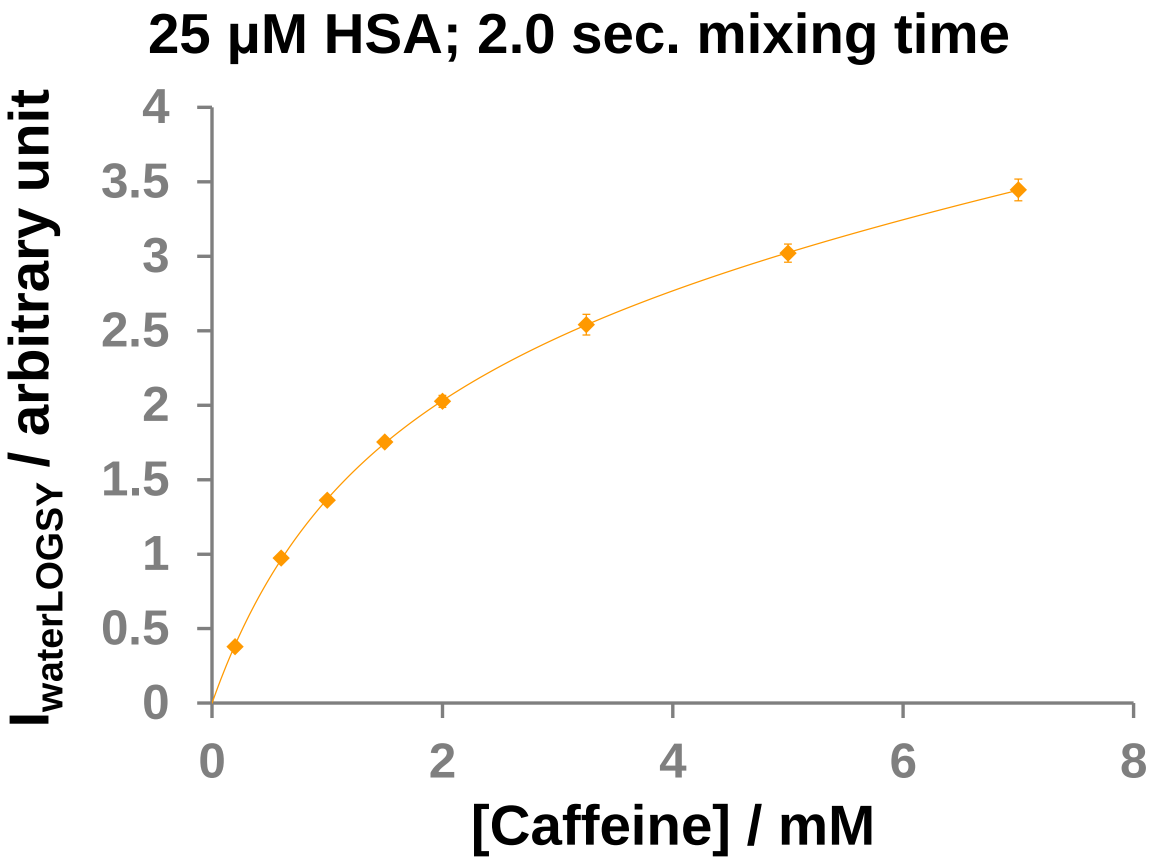

Supplementary Figure S34: Non-linear curve fitting to a corrected waterLOGSY binding isotherm. The mixing time was 2.0 seconds and the relaxation delay was 15 seconds. The system contained 25 µM human serum albumin (HSA) and 0.2–7.0 mM caffeine (binder) in 50 mM sodium phosphate buffer (pH 7.5) at 90% H_2O and 10% D_2O. The fitted K_D^{obs} was 1.5 mM ± 0.2 mM. The errors shown are the standard error from three separate measurements.

(a)

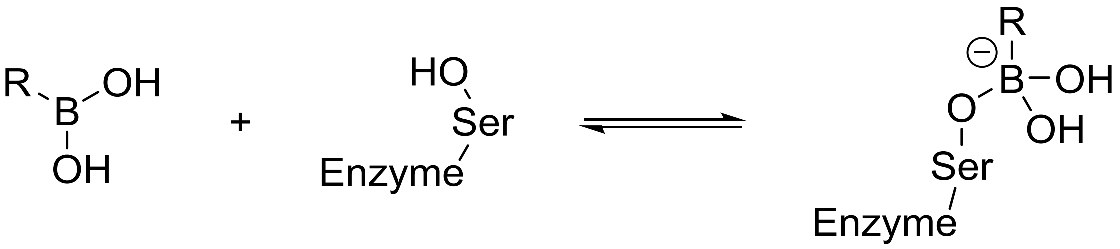

(b)

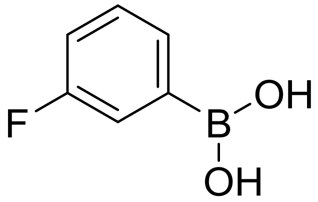

3-fluorophenylboronic acid

Supplementary Figure S35: (a) α-Chymotrypsin, a serine protease, forms a reversible 1:1 covalent complex with boronic acid; (b) Structure of 3-fluorophenylboronic acid that was used as a model ligand system in this study. The doublet resonance of H6 was used for waterLOGSY analysis.

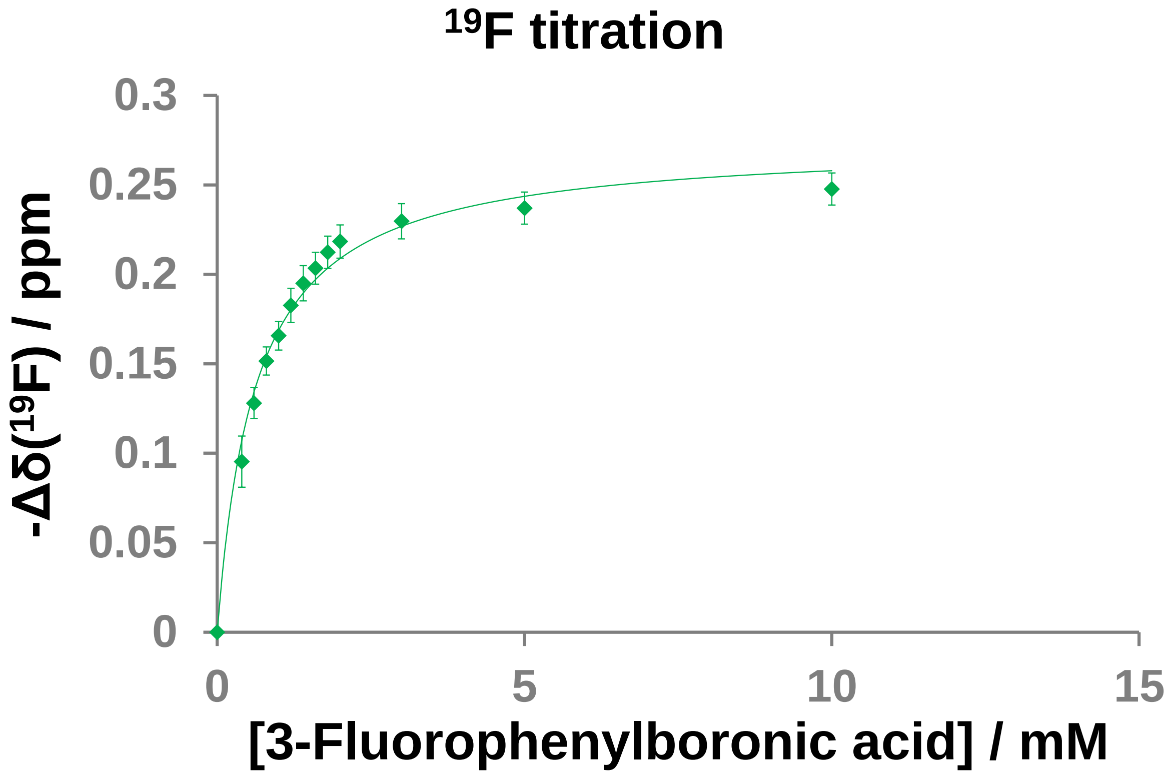

Supplementary Figure S36: Non-linear curve fitting to a binding isotherm between α-chymotrypsin and 3-fluorophenylboronic acid by following changes in ^{19}F chemical shift. Samples contained 100 µM α-chymotrypsin and varying concentrations of 3-fluorophenylboronic acid (stock solution prepared in DMSO-D6) buffered in 100 mM MES (pH 6.5) in 90% H_2O and 10% D_2O. A trace of trifluoroacetone was added to the sample for chemical shift reference at -87 ppm. The fitted K_D was 630 µM ± 60 µM. The errors shown are the standard error from three separate sample preparations.

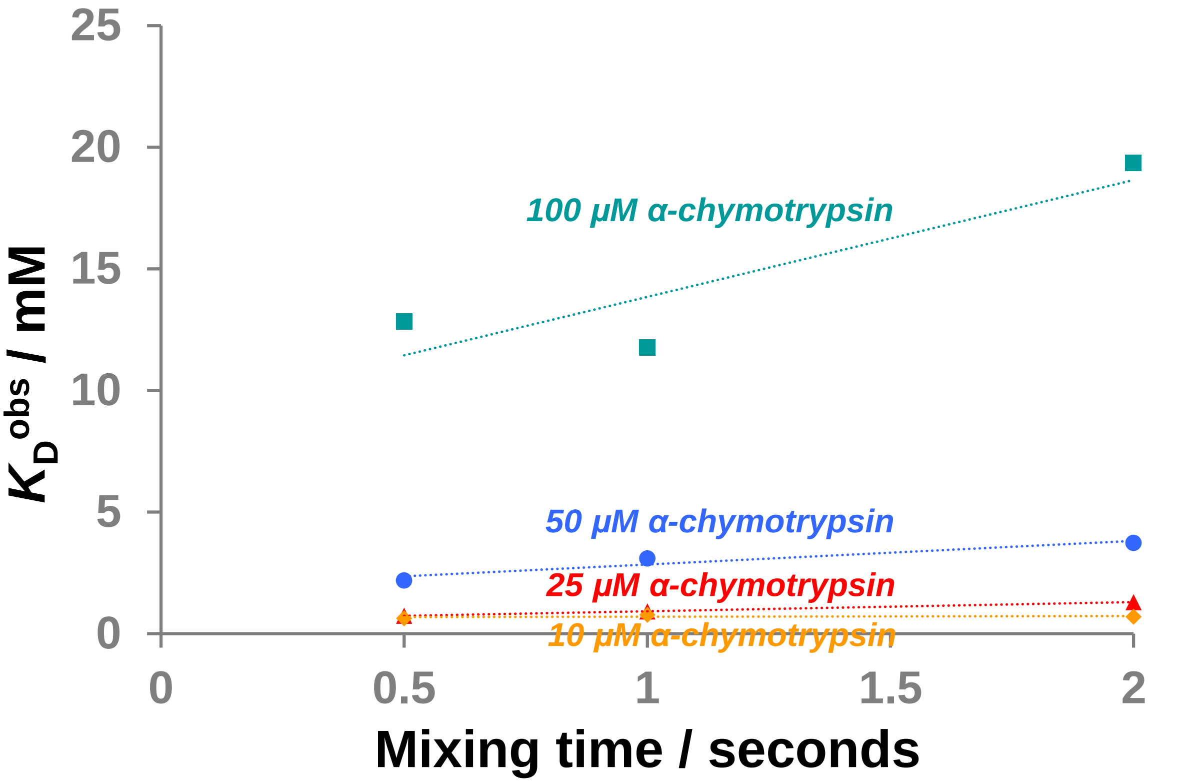

	10 μM α-chymotrypsin	25 μM α-chymotrypsin	50 μM α-chymotrypsin	100 μM α-chymotrypsin
Mixing time / s	K_D^{obs} / mM	K_D^{obs} / mM	K_D^{obs} / mM	K_D^{obs} / mM
0.5	~0.63	~0.74	~2.2	~13
1.0	~0.78	~0.92	~3.1	~12
2.0	~0.70	~1.3	~3.7	~19

Supplementary Figure S37: The correlation between observed K_D (K_D^{obs}), mixing time and α-chymotrypsin concentration for 3-fluorophenylboronic acid binding. The dotted lines are added to aid visualisation.

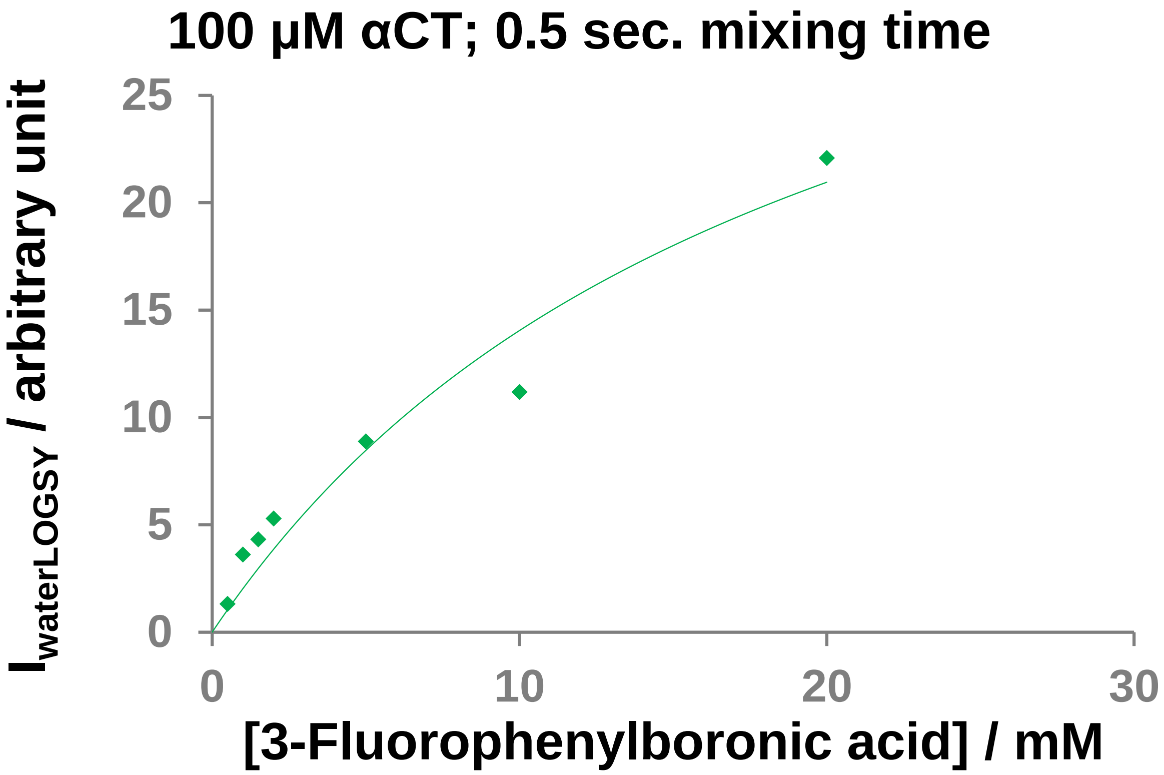

Supplementary Figure S38: Non-linear curve fitting to a corrected waterLOGSY binding isotherm. The mixing time was 0.5 seconds and the relaxation delay was 3 seconds. The system contained 100 µM α-chymotrypsin and varying concentrations of 3-fluorophenylboronic acid (binder) in 100 mM MES buffer (pH 6.5) at 90% H_2O and 10% D_2O. The fitted K_D^{obs} was ~19 mM.

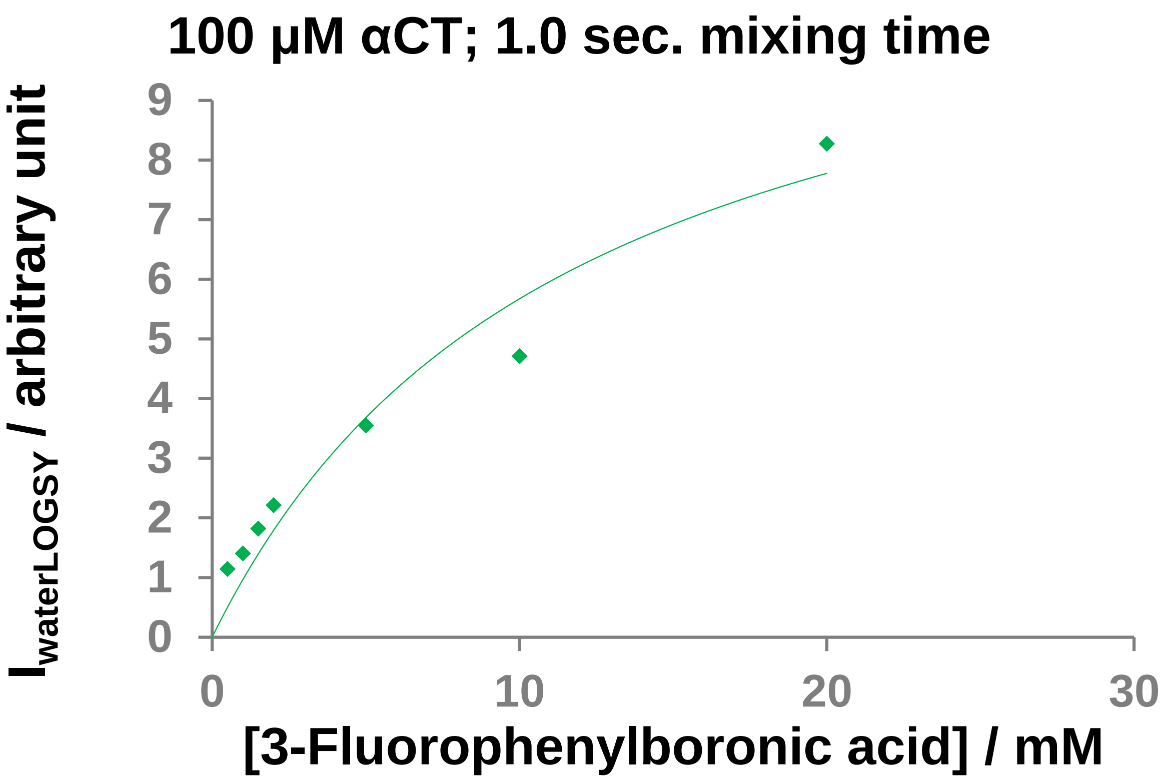

Supplementary Figure S39: Non-linear curve fitting to a corrected waterLOGSY binding isotherm. The mixing time was 1.0 seconds and the relaxation delay was 3 seconds. The system contained 100 µM α-chymotrypsin and varying concentrations of 3-fluorophenylboronic acid (binder) in 100 mM MES buffer (pH 6.5) at 90% H_2O and 10% D_2O. The fitted K_D^{obs} was ~12 mM.

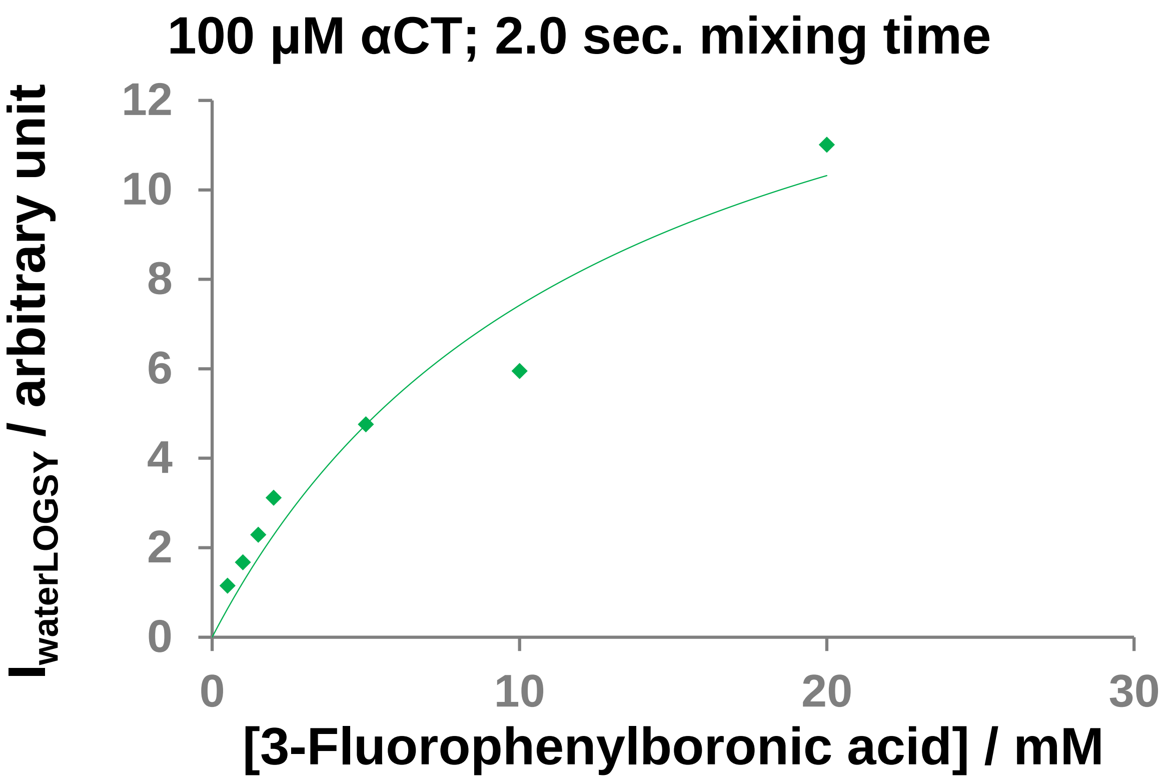

Supplementary Figure S40: Non-linear curve fitting to a corrected waterLOGSY binding isotherm. The mixing time was 2.0 seconds and the relaxation delay was 3 seconds. The system contained 100 µM α-chymotrypsin and varying concentrations of 3-fluorophenylboronic acid (binder) in 100 mM MES buffer (pH 6.5) at 90% H_2O and 10% D_2O. The fitted K_D^{obs} was ~13 mM.

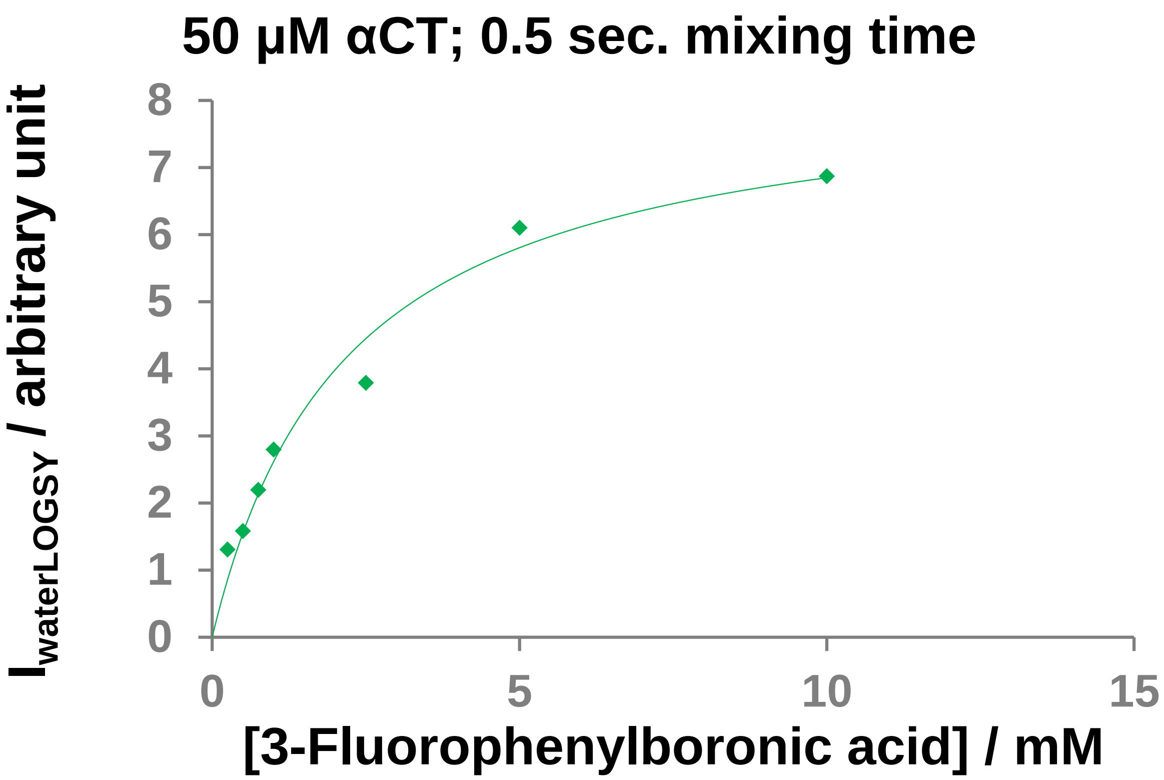

Supplementary Figure S41: Non-linear curve fitting to a corrected waterLOGSY binding isotherm. The mixing time was 0.5 seconds and the relaxation delay was 3 seconds. The system contained 50 µM α-chymotrypsin and varying concentrations of 3-fluorophenylboronic acid (binder) in 100 mM MES buffer (pH 6.5) at 90% H_2O and 10% D_2O. The fitted K_D^{obs} was ~2.2 mM.

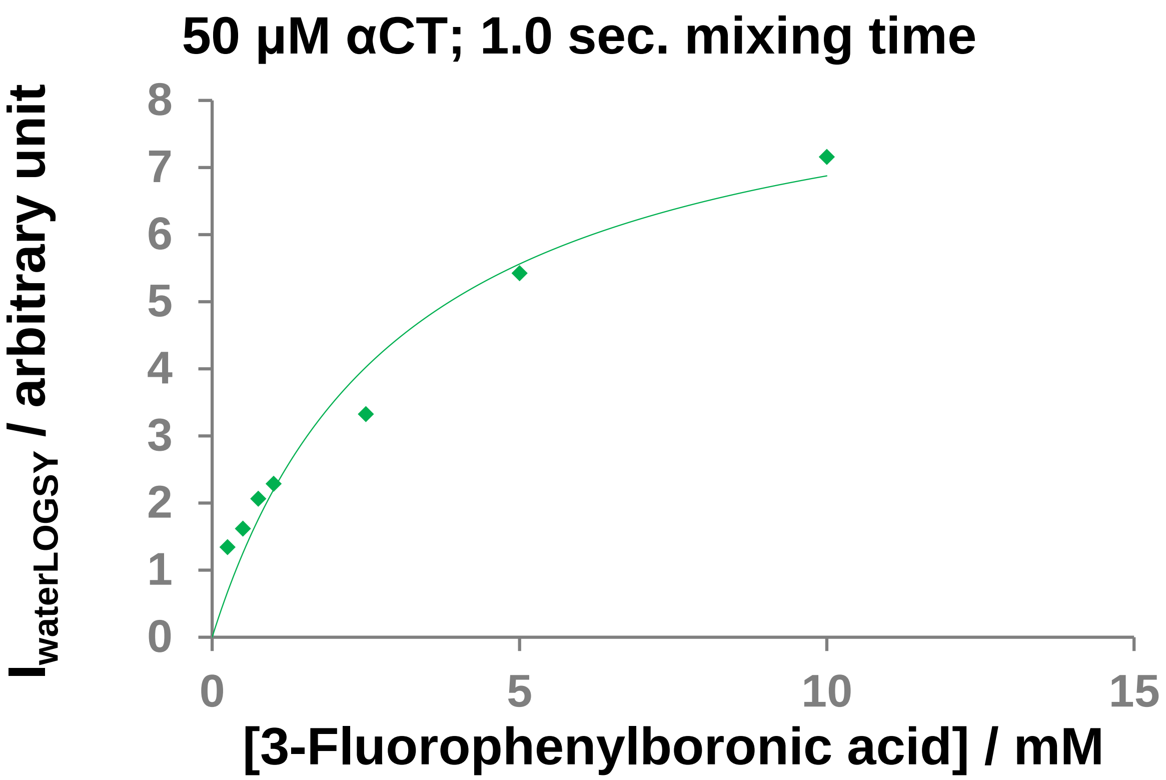

Supplementary Figure S42: Non-linear curve fitting to a corrected waterLOGSY binding isotherm. The mixing time was 1.0 seconds and the relaxation delay was 3 seconds. The system contained 50 µM α-chymotrypsin and varying concentrations of 3-fluorophenylboronic acid (binder) in 100 mM MES buffer (pH 6.5) at 90% H_2O and 10% D_2O. The fitted K_D^{obs} was ~3.1 mM.

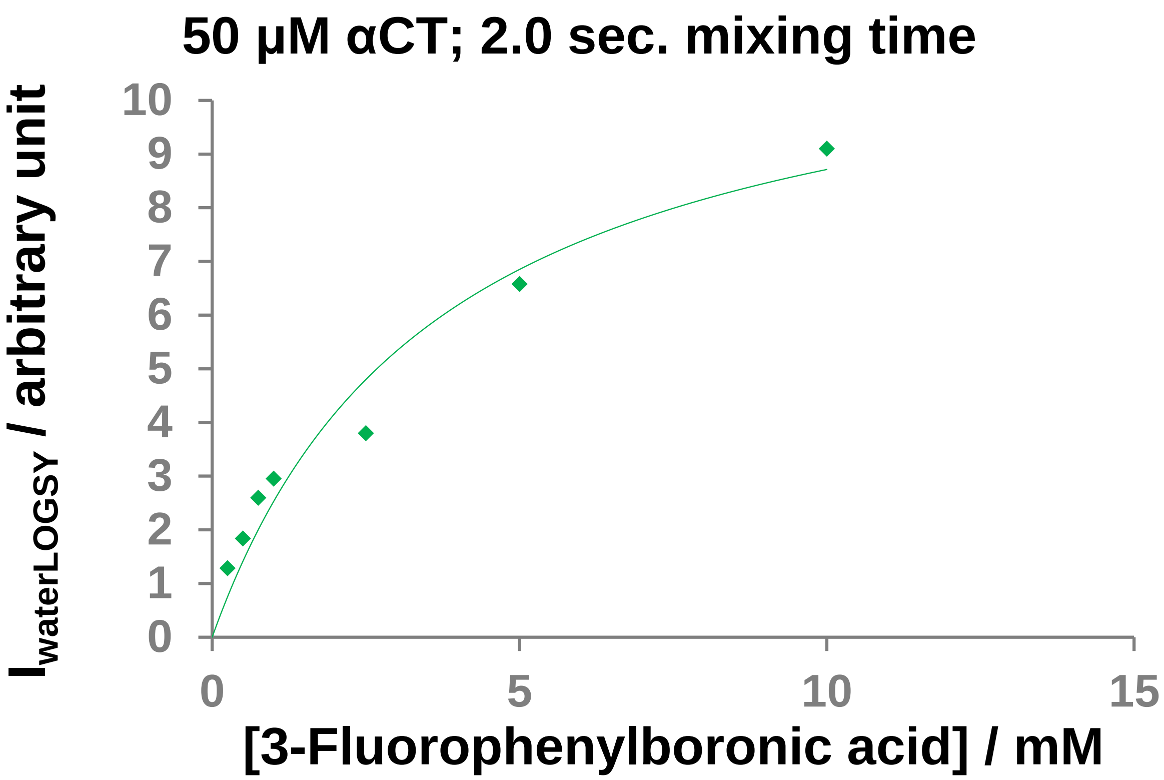

Supplementary Figure S43: Non-linear curve fitting to a corrected waterLOGSY binding isotherm. The mixing time was 2.0 seconds and the relaxation delay was 3 seconds. The system contained 50 µM α-chymotrypsin and varying concentrations of 3-fluorophenylboronic acid (binder) in 100 mM MES buffer (pH 6.5) at 90% H_2O and 10% D_2O. The fitted K_D^{obs} was ~3.7 mM.

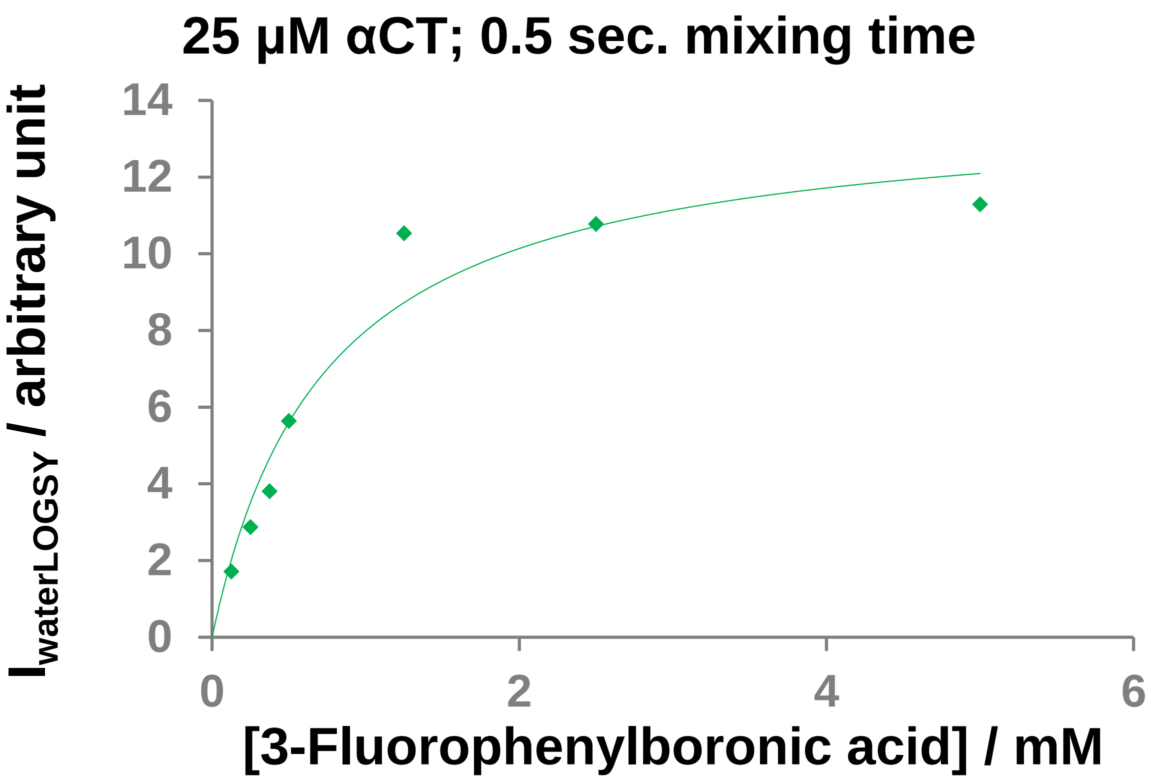

Supplementary Figure S44: Non-linear curve fitting to a corrected waterLOGSY binding isotherm. The mixing time was 0.5 seconds and the relaxation delay was 3 seconds. The system contained 25 µM α-chymotrypsin and varying concentrations of 3-fluorophenylboronic acid (binder) in 100 mM MES buffer (pH 6.5) at 90% H_2O and 10% D_2O. The fitted K_D^{obs} was ~740 µM.

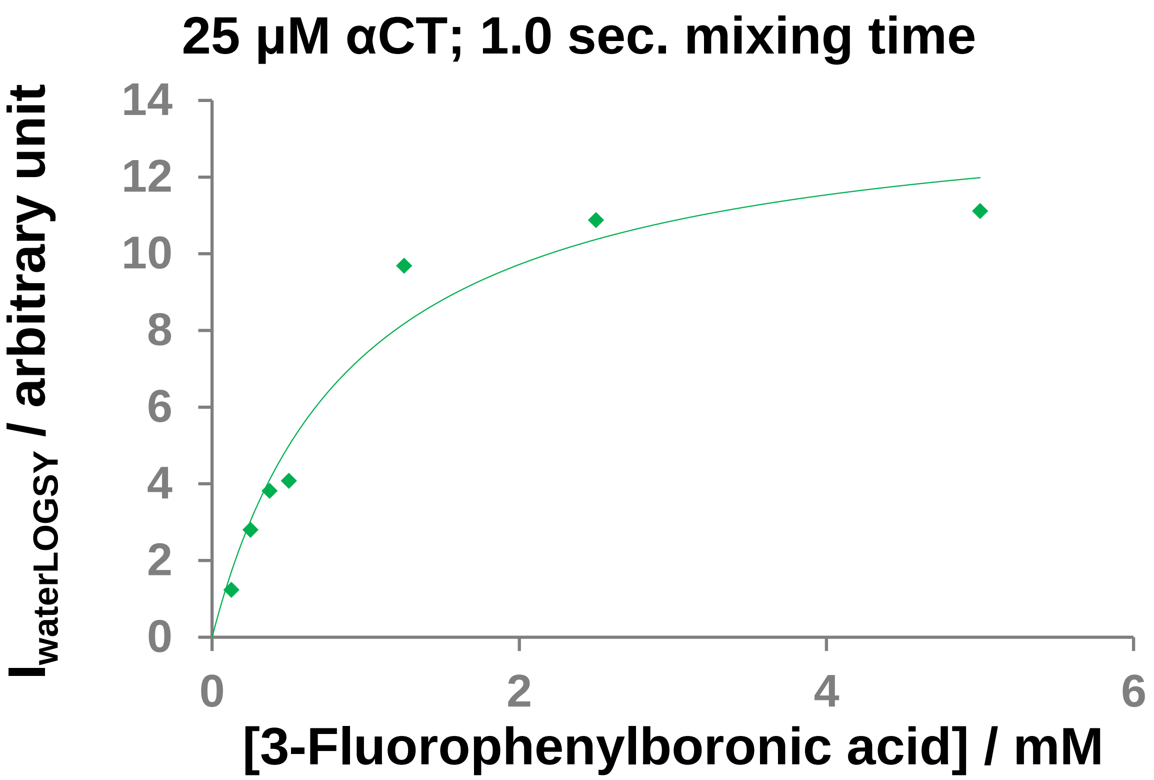

Supplementary Figure S45: Non-linear curve fitting to a corrected waterLOGSY binding isotherm. The mixing time was 1.0 seconds and the relaxation delay was 3 seconds. The system contained 25 µM α-chymotrypsin and varying concentrations of 3-fluorophenylboronic acid (binder) in 100 mM MES buffer (pH 6.5) at 90% H_2O and 10% D_2O. The fitted K_D^{obs} was ~920 µM.

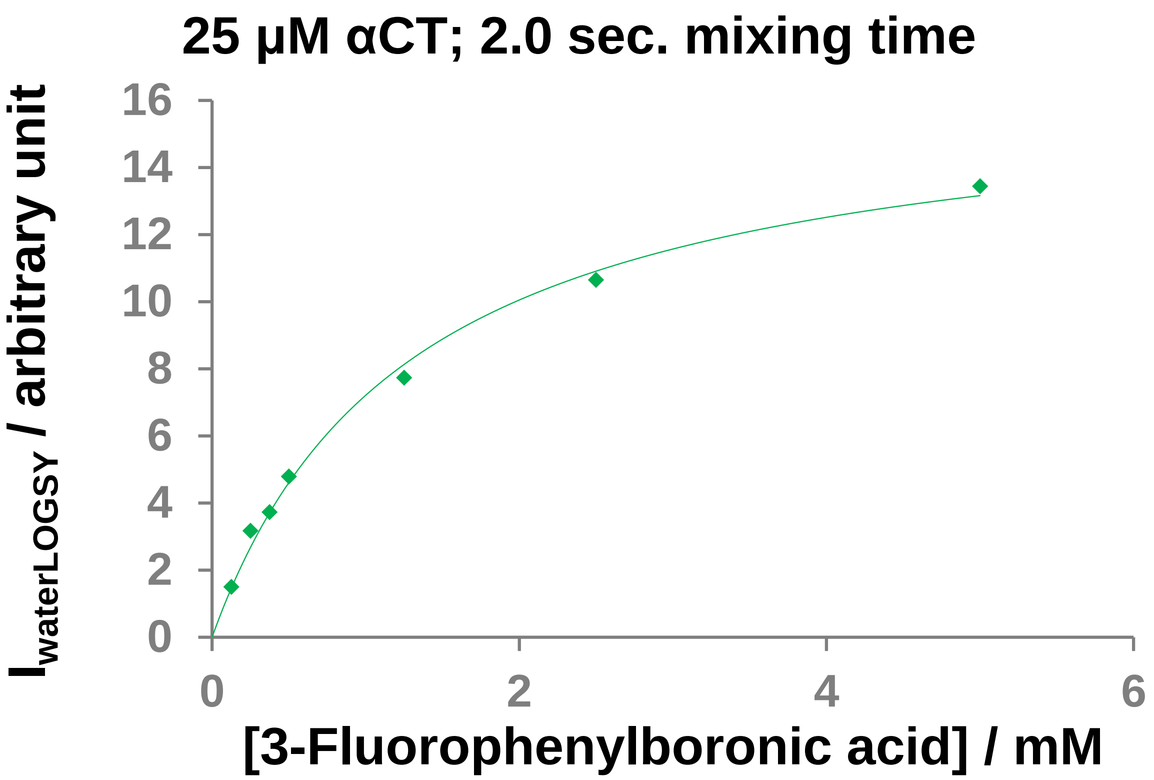

Supplementary Figure S46: Non-linear curve fitting to a corrected waterLOGSY binding isotherm. The mixing time was 2.0 seconds and the relaxation delay was 3 seconds. The system contained 25 µM α-chymotrypsin and varying concentrations of 3-fluorophenylboronic acid (binder) in 100 mM MES buffer (pH 6.5) at 90% H_2O and 10% D_2O. The fitted K_D^{obs} was ~1.3 mM.

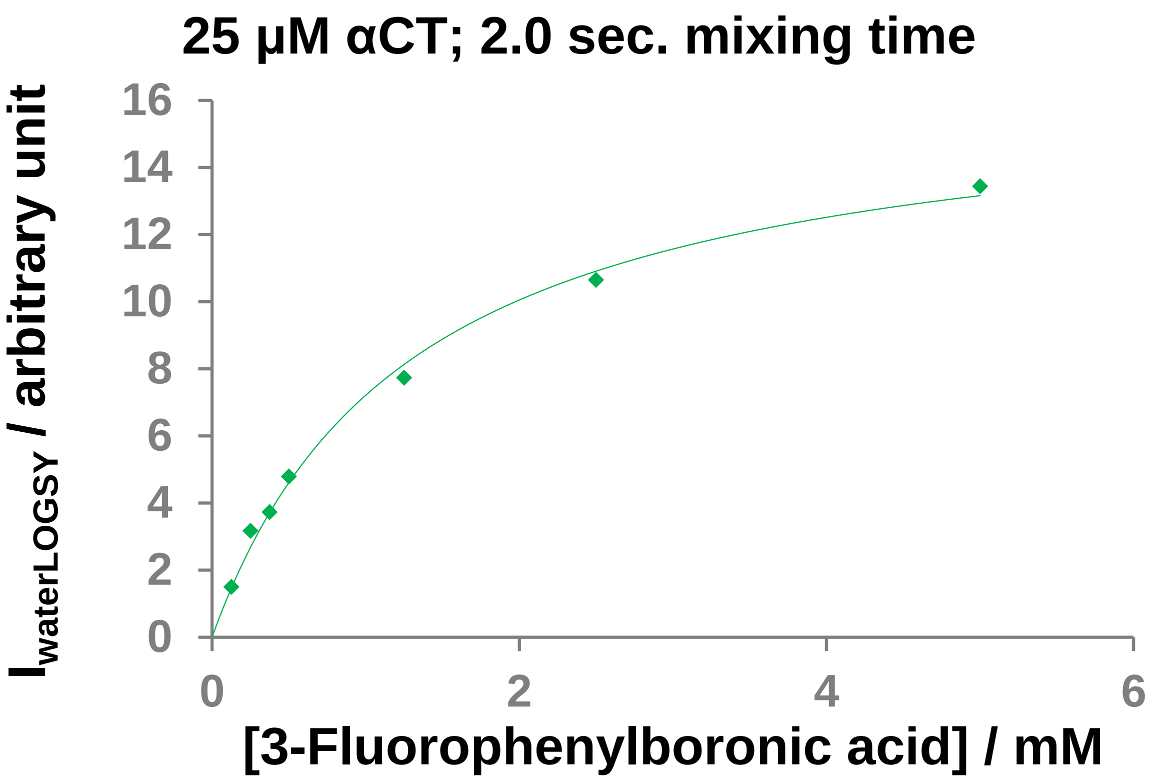

Supplementary Figure S47: Non-linear curve fitting to a corrected waterLOGSY binding isotherm. The mixing time was 0.5 seconds and the relaxation delay was 3 seconds. The system contained 10 µM α-chymotrypsin and varying concentrations of 3-fluorophenylboronic acid (binder) in 100 mM MES buffer (pH 6.5) at 90% H_2O and 10% D_2O. The fitted K_D^{obs} was ~630 µM.

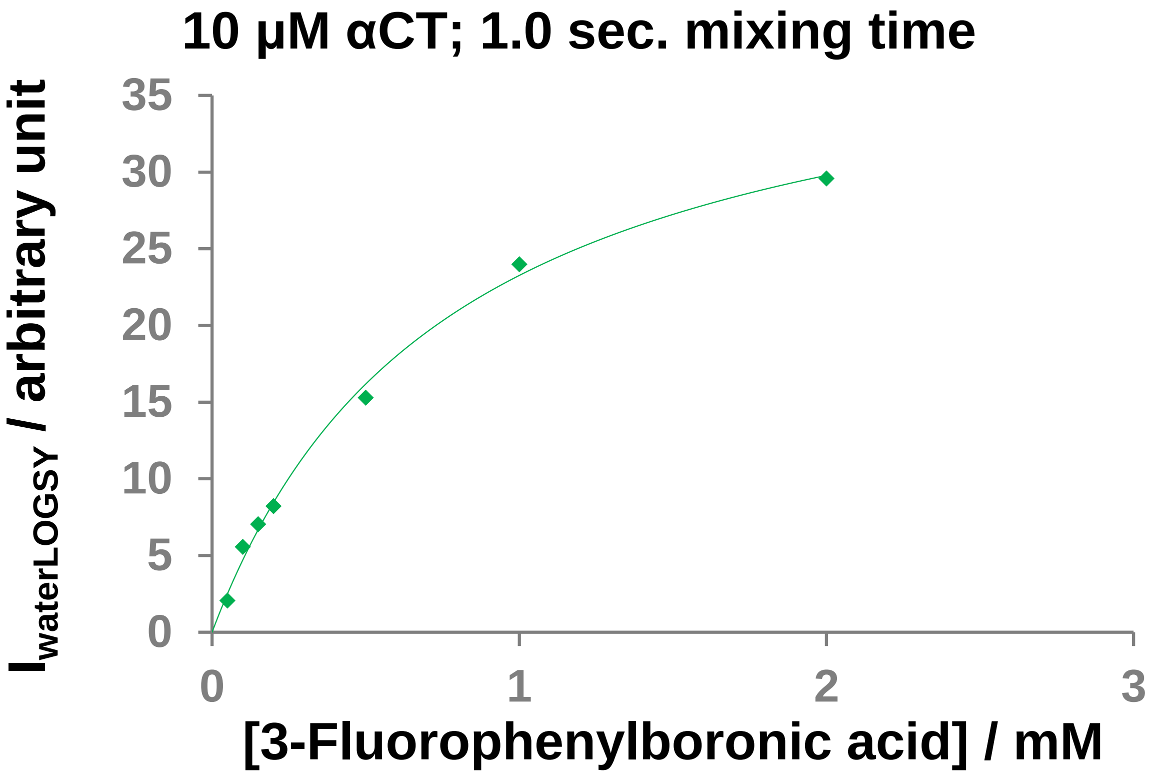

Supplementary Figure S48: Non-linear curve fitting to a corrected waterLOGSY binding isotherm. The mixing time was 1.0 seconds and the relaxation delay was 3 seconds. The system contained 10 µM α-chymotrypsin and varying concentrations of 3-fluorophenylboronic acid (binder) in 100 mM MES buffer (pH 6.5) at 90% H_2O and 10% D_2O. The fitted K_D^{obs} was ~780 µM.

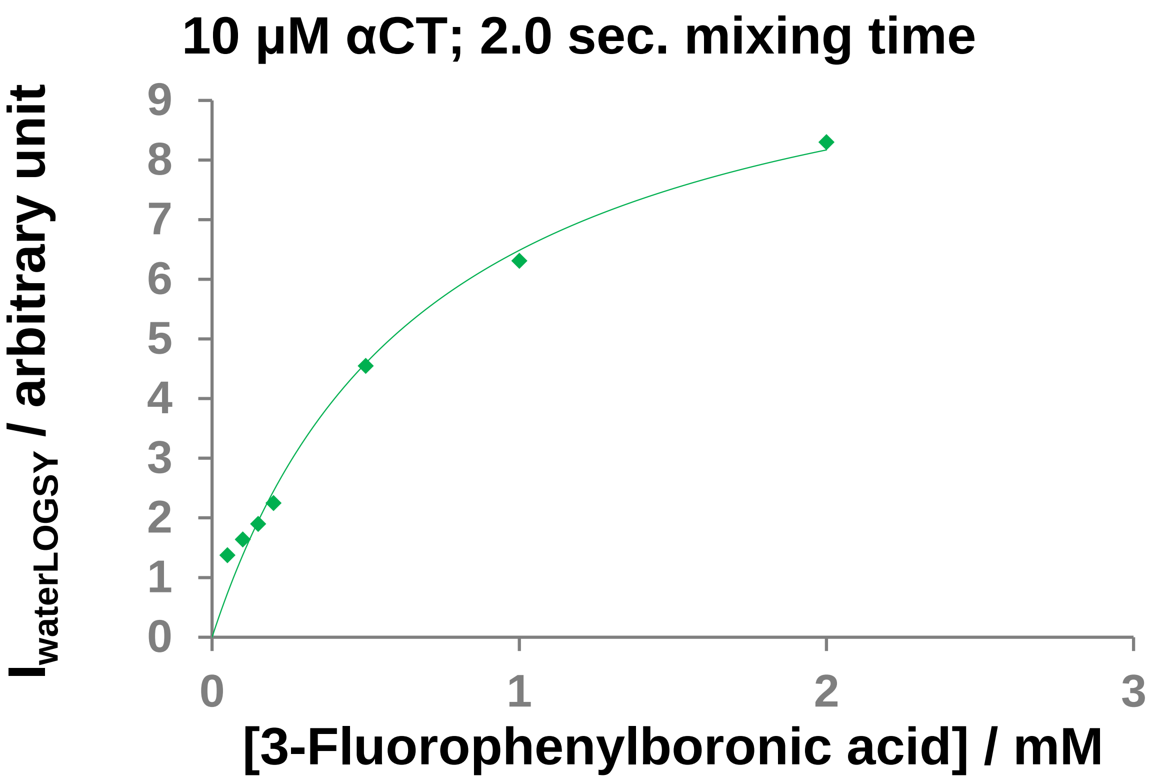

Supplementary Figure S49: Non-linear curve fitting to a corrected waterLOGSY binding isotherm. The mixing time was 2.0 seconds and the relaxation delay was 3 seconds. The system contained 10 µM α-chymotrypsin and varying concentrations of 3-fluorophenylboronic acid (binder) in 100 mM MES buffer (pH 6.5) at 90% H_2O and 10% D_2O. The fitted K_D^{obs} was ~700 µM.
